# Efficient Hybrid Particle-Field Coarse-Grained Model of Polymer Filler Interactions: Multiscale Hierarchical Structure of Carbon Black Particles in Contact with Polyethylene

**DOI:** 10.1021/acs.jctc.0c01095

**Published:** 2021-02-12

**Authors:** Stefano Caputo, Velichko Hristov, Antonio De Nicola, Harald Herbst, Antonio Pizzirusso, Greta Donati, Gianmarco Munaò, Alexandra Romina Albunia, Giuseppe Milano

**Affiliations:** † Dipartimento di Chimica e Biologia, Università di Salerno, Via Giovanni Paolo II, 132, I-84084, Fisciano, Salerno, Italy; ‡ Innovation & Technology, Borealis Polyolefine GmbH, St.-Peter-Straße 25, 4021, Linz, Austria; § Department of Organic Materials Science, Yamagata University, 4-3-16 Jonan, Yonezawa, Yamagata-ken 992-8510, Japan; ∥ Dipartimento di Scienze Matematiche e Informatiche, Scienze Fisiche e Scienze della Terra, Università degli Studi di Messina, Viale F. Stagno d’Alcontres 31, 98166 Messina, Italy

## Abstract

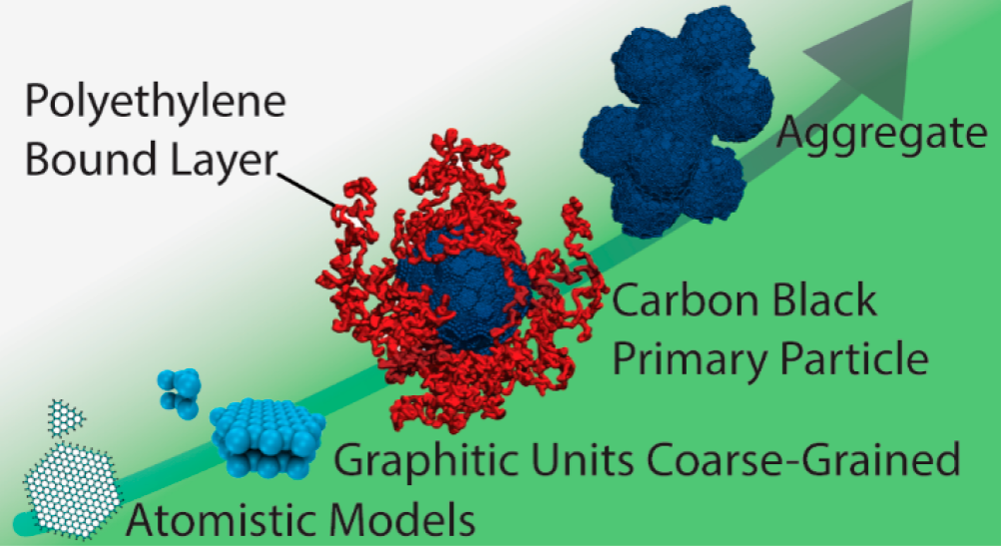

In the present study, we propose, validate, and give first applications for large-scale systems of coarse-grained models suitable for filler/polymer interfaces based on carbon black (CB) and polyethylene (PE). The computational efficiency of the proposed approach, based on hybrid particle-field models (hPF), allows large-scale simulations of CB primary particles of realistic size (∼20 nm) embedded in PE melts. The molecular detailed models, here introduced, allow a microscopic description of the bound layer, through the analysis of the conformational behavior of PE chains adsorbed on different surface sites of CB primary particles, where the conformational behavior of adsorbed chains is different from models based on flat infinite surfaces. On the basis of the features of the systems, an optimized version of OCCAM code for large-scale (up to more than 8 million of beads) parallel runs is proposed and benchmarked. The computational efficiency of the proposed approach opens the possibility of a computational screening of the bound layer, involving the optimal combination of surface chemistry, size, and shape of CB aggregates and the molecular weight distribution of the polymers achieving an important tool to address the polymer/fillers interface and interphase engineering in the polymer industry.

## Introduction

Particulate fillers are powders made of small particles (usually <100 μm) added at different loadings to polymer formulations.^1^ These fillers are largely used in the polymer industry with the aim of reducing costs or tailoring and improving materials performances.^1−8^ They can be mineral based particulate fillers such as carbonates (principally calcium carbonates), kaolins, mikas, talcs, or synthetic particulate fillers such as CB and precipitated (or fumed) silicas.^1^ CB and other more expensive carbonaceous fillers are widely employed to improve the properties of polymeric materials. Main fields of application of CB polymer composites are, for example, tires, rubber, wire and cables, and pressure pipes. CB fillers influence thermal, conductive, antistatic, and dissipative properties, with an impact on the coloration, UV resistance, and electromagnetic properties too.^1^ Moreover, CB is the most common conductive filler for composites based on different types of PE.^9^ At a given concentration, the conductive properties of CB/PE composites depend on the physical–chemical characteristics of CB and its dispersion in the final product, and in particular, it turns out to be dependent on the polymer–filler interactions and on the compounding conditions.^9^

In order to gain knowledge into these phenomena, a deep understanding of the interaction between polymer and filler is needed. Many factors act at different length scales: the chemistry of the surface, the surface roughness, and the size and curvature of the filler particles. All of them influence polymer–filler interactions. Moreover, to describe these interactions, it is necessary to characterize the interface and interphases formed in the filler’s surroundings. Several experimental studies, over the past three decades, based on rheological properties,^10,11^ nuclear magnetic resonance (NMR),^12−14^ and small angle neutron scattering (SANS),^15^ converge on the picture of a distinct interfacial bound layer of polymer chains extending over the nanometer length scale from the filler surface. As proposed earlier by Stickney and Falb for rubber,^16^ this bound layer is a stabilized film around the particles thus being resistant to dissolution in solvent.^14,17^ The concept of the bound layer is generally accepted, and several experiments and theoretical schemes for the interpretation of observed experimental trends have been conducted to characterize its properties.^15,18−21^

On the molecular scale, local interactions between atoms or small groups of atoms belonging to both polymer chains and the surface, largely determine the structure of chain segments close to a solid surface. Because of the short-range nature of the adsorption interactions and the low number of the polymer’s repeating units involved, the fraction of the chain segments strongly influenced by the surface is small. Therefore, a direct experimental investigation of polymer chains at the surfaces is, in practice, rather limited. Many experimental studies in this direction focused on different rubbers filled with CB.^19,22−25^ A quantitative indirect evaluation of the amount of polymer bound around the filler is based on simple solubilization experiments and has been achieved in early studies on this subject.^15,16,26^ More difficult is the direct evaluation of different properties of the bound layer in polymer composites for the small predicted thickness on the nanometer scale. NMR experiments suggested the existence of an interphase, in which chain mobility near the CB–rubber interfaces is less than that of the bulk polymer.^14,27−29^ More recently, the bound polymer layer interphase has been visualized in cured hydrogenated nitrile butadiene rubber/CB composites using scanning probe microscopy (SPM) analysis.^30^ In this study, the visualization of this particle–polymer interphase has been conducted by atomic force microscopy (AFM) phase imaging.^30^

**Figure 1:**
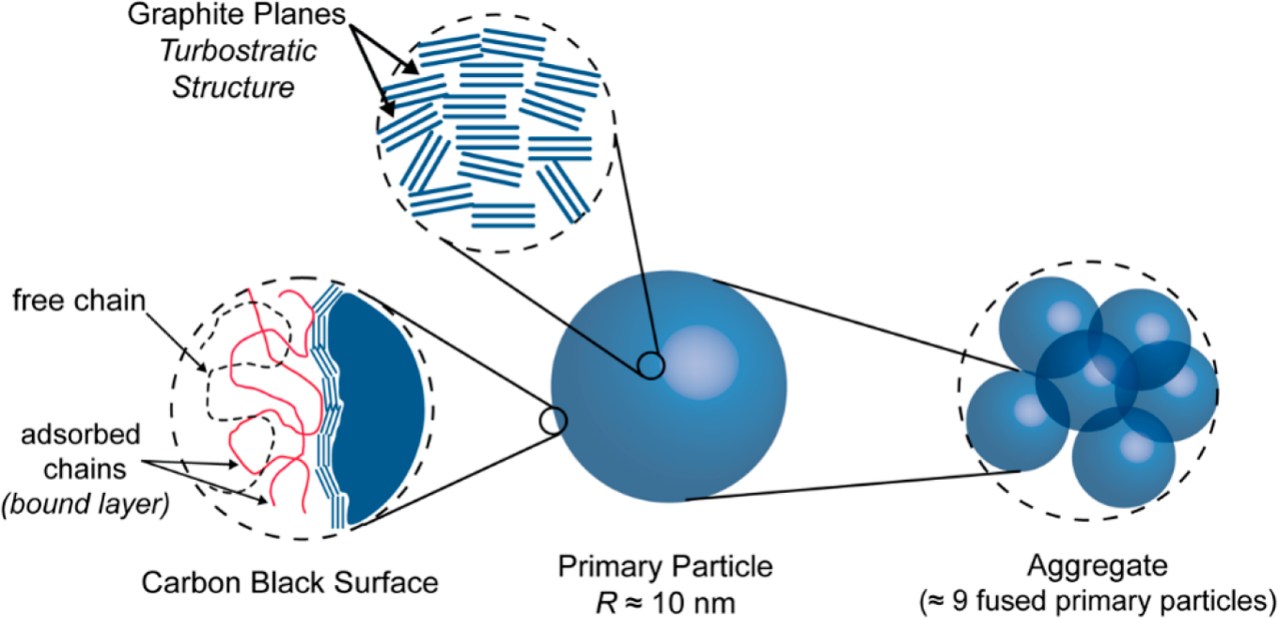
Graphical sketch of the hierarchical structure of CB fillers.

The difficulty to obtain a direct characterization of the bound layer having a thickness in the nanometer range, and the lack of detailed molecular scale experimental information on this interphase, made molecular simulations very appealing. Several atomistic molecular dynamics (MD) simulation studies have been focused on the clarification of the structure and dynamics of nanoparticles (NPs) and solid surfaces in contact with polymers.^31−37^ Atomistic simulations provide an accurate description of the interphase, but they are typically limited to small polymer chain lengths, small nanoparticles (typically with a radius of few nanometers or represented by idealized infinite and regular surfaces in short sized periodic boundary conditions) and short time scales. This limitation is very strong if we consider fillers having large size and a complex hierarchical structure spanning several length-scales such as CB (see Figure 1).

As described in Figure 1, CB fillers are commonly specified by different characteristic sizes. Starting from the smallest scale (atomic or molecular, 1 nm or less), according to the results of adsorption experiments,^38^ the surface of CB is characterized by different adsorption sites. CB aggregates are spherical arrangements of microcrystallites with a size distribution centered around a radius of ∼10 nm.^17,24,39^ CB fillers in polymer composites are not present as dispersed graphitic units, not even as single primary particles. On the contrary, several primary particles (on average 9–10) are arranged as aggregates (with a radius of ∼30 nm) and commonly two or four of these aggregates form the smallest dispersible units in polymeric materials. The miscibility and the degree of dispersion in polymers are strongly related not only to the surface properties of CB (like the presence of high energy surface sites) but also to the size of the primary particles, the aggregate morphology of a given CB grade, and the compounding conditions. This is reasonable because, considering the length scales of a typical polymer chain ranging from a single repeating unit (<1 nm) to the persistence length to the radius of gyration of an entire chain (30–100 nm), an interplay between CB and polymer structure can be expected at different levels. With this in mind, it is clear how a model able to take into account features from molecular scale up to a realistic aggregate size (∼100 nm) would be highly desirable.

Recent advances on synthesis and characterization methods applied to CB filler technology are opening the way to a rational design of materials and processing conditions. In particular, to the shortest scale, the surface chemistry of CB corresponds to that of graphitic units made of graphene fragments forming its basic structural building blocks. According to this, novel methods for grafting polymers and functional groups can be easily translated to CB to design and control surface chemistry.^40^ On a larger scale, recent advances in electron microscopy probing the size and shape distributions of CB aggregates have defined multiple descriptor types (size, elongation, ruggedness etc.) assessed for their repeatability and reproducibility. This allows the identification and possibly the design of proper processing conditions of CB aimed to control and to achieve a given aggregate morphology.^41^ In this framework, the contribution of efficient and chemical specific multiscale models to this field would be useful to address filler design on multiple scales.

Several specific coarse-grained (CG) models have been proposed for polymers in the presence of solid particles/surface in order to consider large and properly relaxed systems. Particularly effective to this aim are CG models of NP/polymer interfaces parametrized, on the basis of reference atomistic simulations, using the Iterative Boltzmann Inversion (IBI) method.^42^ These models are able to correctly reproduce the structure for bulk polymers and to describe in close agreement with both atomistic simulations and experiments molecular details of the structure of polymer interphase around NPs.^43−45^ The reduction of degrees of freedom operated in CG models, needed due to the large size of the systems, is not enough to handle a proper description of CB as an aggregate of primary particles and not even as a single primary particle in contact with a polymer melt, although it allows an efficient equilibration of polymer melts also in contact with a solid surface. Indeed, also in the most recent literature, the presence of CB fillers is still modeled using CG models of a regular (no surface sites) planar infinite surface. For example, very recently, Giunta et al. modeled a CB filler in contact with polyisoprene as ∼10 × 10 and ∼20 × 20 nm^2^ regular and planar graphitic surfaces under periodic boundary conditions.^46^ A further speed up of simulations, needed to handle molecular models on the scale required by the CB hierarchical nature, can be obtained combining particle models with density fields. In particular, hPF MD, based on a combination of MD with self consistent field (SCF) theory,^47,48^ is more suitable to this aim. The hPF MD simulations have been successfully applied to polymer nanocomposites to obtain well relaxed systems, interphase structures, free energy for silica NP separation in specific models of monodisperse and bidisperse polystyrene melts, and interfaces between block copolymers and grafted solid surfaces.^49−53^ Simulation results compare well with available experimental data regarding interphase structures and the stability of the microscopic structures of the composites in a wide range of conditions. More recently, Theodorou and co-workers studied the behavior of a PE melt onto a planar and regular graphitic substrate using a methodology based on SCF theory parametrized from atomistic molecular simulations.^54^

In the present study, we propose, validate, and give first applications of a hPF CG model suitable for CB/PE interfaces and interphases parametrized from atomistic simulations. Large-scale models of a core–shell CB primary particle embedded in PE melts are described, and a detailed molecular characterization of the conformational behavior of PE chains adsorbed on CB primary particle surfaces, in comparison with a flat graphitic surface, is provided. The paper structure is described as follows: In the Methods and Models section, a brief overview of hPF MD methodology and a description of the code optimizations are given together with a description of mapping schemes for the different CG models introduced. The Results and Discussion section is divided into different parts. The first and the second parts describe PE CG simulations of melts, their validation against atomistic simulations, experiments and the tuning of interaction between PE and the graphitic surface. The third part is about the CB primary particle model and its validation. The fourth part reports the results of parallel simulations (ranging from ∼200 000 to ∼500 000 beads) of CB/PE interphases. Finally, the last part describes benchmarks of an optimized version of the OCCAM code suitable for parallel runs for large scale systems (up to 8.4 million of beads) modeling polymer–filler interactions. Conclusions and Perspectives follow in the last section.

## Methods and Models

### Hybrid Particle-Field Molecular Dynamics Simulations of CG Models

**Chart 1:**
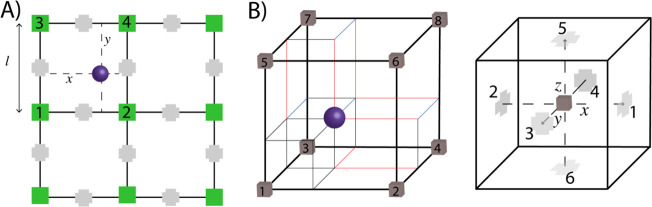
(A) Particle Projection Scheme on a Grid in a Two-Dimensional Examplea and (B) Particle Fraction Assignment for the Case of the Three-Dimensional Lattice Employed in the Simulations, Gradients Defined on a Staggered Latticeb

A complete description and derivation of the simulation approach employed here can be found in refs (47) and (48). As previously mentioned, the hybrid particle-field approach applied in the present work has been validated and widely employed in several previous works to get equilibrated polymer melts and models of polymer composite materials at both all-atom and coarse-grained levels.^50,52,55−57^ The main advantage of hybrid particle-field models is that the evaluation of nonbonded forces and potentials is obtained through the calculation of an external potential depending on the local density at the particle position *V*(***r***). It can be shown that, knowing a given functional form of the free energy and the external potential acting on a particle of type *K* at position ***r***, *V*_*K*_(***r***) can be written as the functional derivative of total energy *W*[ρ_*K*_(***r***)]:
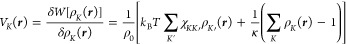
1awhere
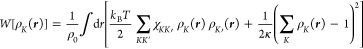
1bIn particular, in eq 1a, *k*_B_ is the Boltzmann constant, *T* is the temperature, ρ_*K*_(***r***) is the number density of the species *K* at position ***r***, χ_*KK*′_ are mean-field parameters for the interaction of a particle of type *K* with the density fields due to particles of type *K′*, and κ is the compressibility. More details about the hybrid approach and a complete derivation of eq 1 are reported in refs (47) and (48). To connect the particles and the field, a smooth *coarse-grained* number density field ρ_*K*_(***r***) is obtained starting from the position of the particles. To this aim, as schematized in Chart 1, a mesh-based approach is applied; the simulation box is divided into subcells, and according to the particle positions, a fraction of number density is assigned to each vertex of the subcell.

a The particle fraction assigned to a given vertex is proportional to the area of a rectangle whose diagonal is the line connecting the particle position and the mesh point on the opposite side of the cell. b the density gradients are defined on the center of each face.

In this mesh-based scheme, the coarseness of the density is tuned by the mesh size *l*. A frequency Δ*t*_update_ is set to update the density field from a new coordinate set at time *t* + Δ*t*. For all simulations reported herein, the frequency update Δ*t*_update_ was set to 3 ps analogously to the value used for similar CG models employed in previous studied systems.^52^ The mesh size *l* in all reported simulations was set to 0.73 nm (approximately one bond length in the CG model). To calculate forces due to the density fields, spatial derivatives of the external potential (eq 1a) are computed from the finite difference of the mesh where the density field is calculated on the basis of particle positions. The time step for the velocity Verlet algorithm for particle displacement was set to 0.03 ps. Simulations have been run in the NVT ensemble; temperature is controlled by an Andersen Thermostat using a collision frequency of 7 ps^–1^.

### Interface Mode Algorithm

**Chart 2:**
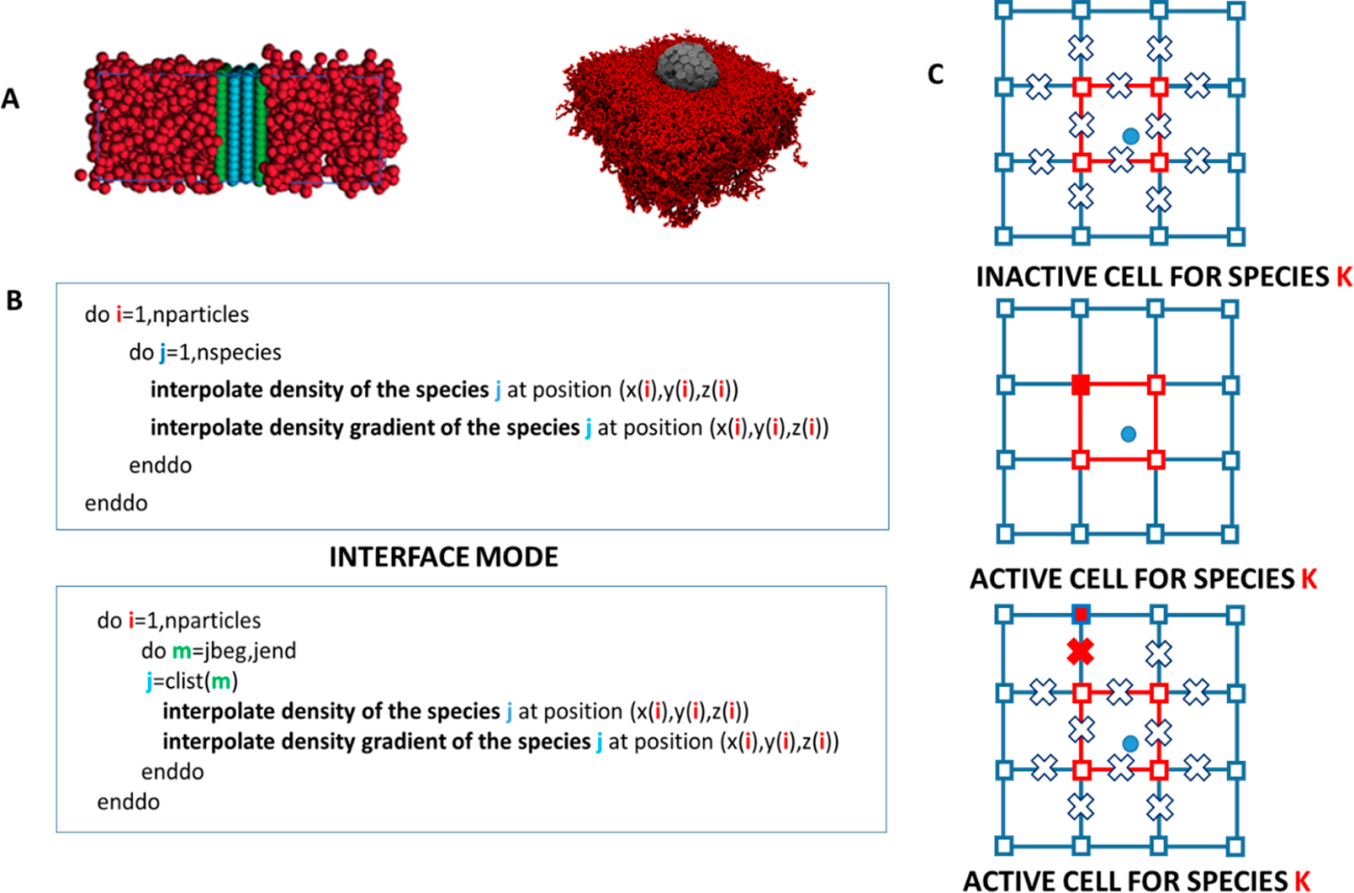
(A) Schematization of Simulated Systems, (B) Pseudocodes for Standard (Upper Panel) and Optimized (Lower Panel) Simulation Code, (C) Scheme for the Assignment of Active and Inactive Species for a Particle (Blue Circle) in a Cell According to the Values of the Density Field (Squares) and Its Derivatives (X Symbol)a

In order to reach suitable simulation times, for the large model systems proposed in this work, an optimized version of the OCCAM code^58^ has been developed. A more efficient calculation of density field derivatives has been implemented. Moreover, the most expensive part of hPF MD calculation, i.e., the interpolation of density field and the density field derivatives at the particle position, has improved to save computation time. An algorithm named Interface Mode (IM) for a more efficient calculation of the loop for interpolation has been implemented in the OCCAM code. Although the computational cost of hybrid particle-field simulations is much lower than regular MD simulations employing pairwise nonbonded potentials, it is known that the most expensive part of the hPF MD calculation is the computation of forces due to the density fields.^58^ The interpolation of densities and their spatial derivatives at each particle position for each particle is needed in order to calculate the potential (eq 1a) and the forces. In Chart 2, the pseudocode corresponding to the evaluation of this calculation is reported.

a Parent cell is highlighted in red; neighboring cells are in blue. Filled vertexes and filled X correspond to nonzero values of the density and density derivatives, respectively. Empty symbols correspond to zero values.

The loop needed for these calculations, as reported in the upper panel of Chart 2B, is a double loop over particles and K species corresponding to K particle types of the model (three in the present case). In the case of systems at interfaces, as the ones considered in this study, most of the particles are in contact with one species (the same species K of the particle i), and only the particles located at interfaces are in contact with more species. For both simulated systems, as schematized in Chart 2A, it is clear that most of the polymer beads are in a region in which the density fields of CB particles are absent. Vice versa, in the core of CB particles and in its more internal shells, polymer particles, and hence their field, is not present. According to this, without a loss of accuracy, it is possible to keep track of the species “active” for a given particle, by considering the composition of the cell where the particle is located. This idea has been previously exploited in the GPU implementation of particle-field simulations^59^ but not fully validated and not in the present version. For each particle, it is possible to build a list of “active” species based on the composition of the cell where the particle is located. In order to safely apply this condition, for a given particle, the list of the “active” species can be updated excluding all those K species for which densities are zero in all eight vertexes of the cubic cell and, at the same time, also the density field derivatives (defined on a lattice staggered with respect to the one defined for the density field) are zero. In Chart 2C, some examples of cases of active or inactive species for a given particle are schematized. In particular, the upper panel of Chart 2C shows the case of an inactive species where in all vertexes of the cell, and of the neighboring vertexes (using the condition on the derivative), the density of the species is zero. The middle panel shows a case of active species due to a nonzero value of the density field, while the bottom panel shows a case of active species for a nonzero value of the density derivative. The implementation and the data structure of the list of active species is very close to the one employed to build up the Verlet neighbor list in traditional MD simulations. All the simulations reported in this paper have been run, except the ones reported in the section entitled Large Scale Simulation Benchmarks that were performed for benchmark and validation purposes, using the standard version of OCCAM, then not using IM run modality.

Details about atomistic simulations, the employed force field, and their setup used to parametrize the CG models are reported in the first part of the Supporting Information. In the following subsections, information about CG models of PE, planar graphite (GR), and CB primary particle CG models are reported.

### Polyethylene CG Model

**Figure 2:**
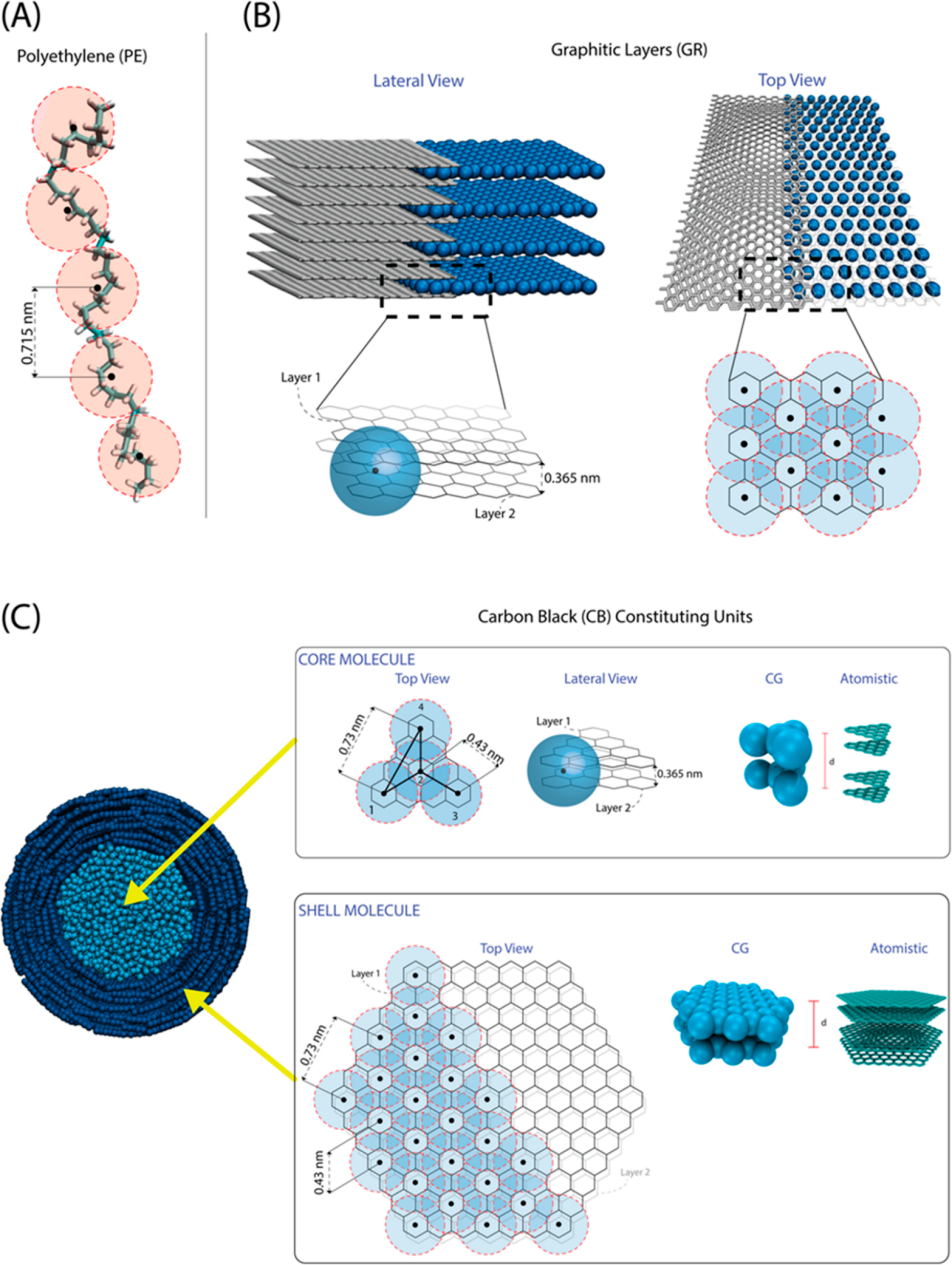
Mapping scheme for CG models for (A) PE. Beads are centered in the center of mass of the corresponding eight carbon atoms of the atomistic model. (B) Regular graphitic surface. Each CG layer corresponds to two atomistic layers; CG beads are centered as indicated by black dots in the figure. (C) Carbon black primary particle, with two different molecules included in the core (core molecule) and in the shell region (shell molecule); the mapping scheme is analogous to the one used for the regular graphitic surface. One CG molecule corresponds to two stacked atomistic molecules as schematized in the top and bottom panels of the figure. In both the upper and lower panels, two stacked CG molecules (corresponding to four atomistic molecules) are shown.

In Figure 2A, the mapping of atoms into CG beads for PE is depicted. In particular, four repeating units (each of two methylene groups for a total of eight carbon atoms and 16 hydrogens) are grouped in one bead. Each bead is centered in the center of mass of the corresponding eight carbon atoms. Intramolecular interactions among beads are modeled by harmonic bond and angle potentials between successive beads described by eqs S9 and S10 in the SI, section 2.6.

As described in the previous subsections, both nonbonded intra- and intermolecular interactions are described through density fields (eq 1). In the case of a homopolymer melt, the only term considered in eq 1 is the incompressibility condition (χ parameter is zero for beads of the same species) with an adjustable parameter κ (the chosen value for 1/κ is 1.0 kJ/mol) depending mainly on the level of CG of the models

### Planar Graphite (GR) CG Model

The mapping scheme for the CG model for graphitic infinite planes is sketched in Figure 2B, where it is shown that each CG bead is centered in the geometric center of the six-membered rings indicated by a black dot in Figure 2B. Moreover, each plane, in the CG representation, corresponds to two planes of carbon atoms, and the center of the beads is placed in the middle of two graphene layers; in this way, each CG bead corresponds to an average of 12 carbon atoms. According to this scheme, we have a short (0.426 nm) and a long (0.738 nm) distance between neighboring beads of the same layer. In Figure 2B, the atomistic model of the graphitic layers is reported together with the corresponding CG model. According to the mapping scheme adopted, the eight layers of the atomistic model used to parametrize the CG model correspond to four layers in CG representation. Bonded and nonbonded interactions among beads belonging to the planar graphite models are not taken into account. Indeed, the CG beads constituting the planar layers are kept frozen during the molecular dynamics simulations by excluding them from the integration algorithm.

### Carbon Black Primary Particle CG Model

Spherical primary particles of CB have been modeled as core–shell structures containing two different molecules; more discussion about this choice is reported in the next section. The mapping scheme of both molecules composing the core and shell layers of the primary particles is very similar to the one used for the planar graphitic model and is reported in Figure 2C. Two different types of bond distances and bond angles are described by harmonic potentials (parameters are reported in the SI) for both CG molecules. Moreover, for the shell molecules, harmonic dihedral potentials are included to keep some planarity (parameters are reported in the SI section). Differently from PE–PE and PE–CB interactions, nonbonded interactions between all molecules belonging to the primary particles are calculated through Lennard-Jones (LJ) pair potentials among CG beads. LJ parameters ϵ and σ have been optimized to reproduce the atomistic interactions between two pairs of planar molecules. Each pair of molecules corresponds to one CG unit as schematized in Figure 2C. Details about parametrization of nonbonded interactions herein used can be found in the Supporting Information section 2.1.

## Results and Discussion

### PE Melt Simulations

Bond and angle parameters have been set to match bond and angle distributions averaged from reference atomistic simulations of PE, and the optimized constants are reported in the Tables S17 and S18 of the SI, section 2.6. Simulated CG systems discussed in this section are listed and described in Table 1; the system name is expressed as PE followed by the number of repeating units equal to 4 times the number of CG beads.

**Table 1:** Simulated Systems: CG Model of PE Melts

system	no. of chains	total no. particles	box size (nm) [*x* = *y* = *z*]	time (μs)	*T* (K)
PE20	191	955	6.145	0.35	423
					468
PE1072	1925	515900	50	5.13	423
					468
PE5880	350	514500	50	18.5	423
					468
PE10696	193	516082	50	16.4	423
					468

**Figure 3:**
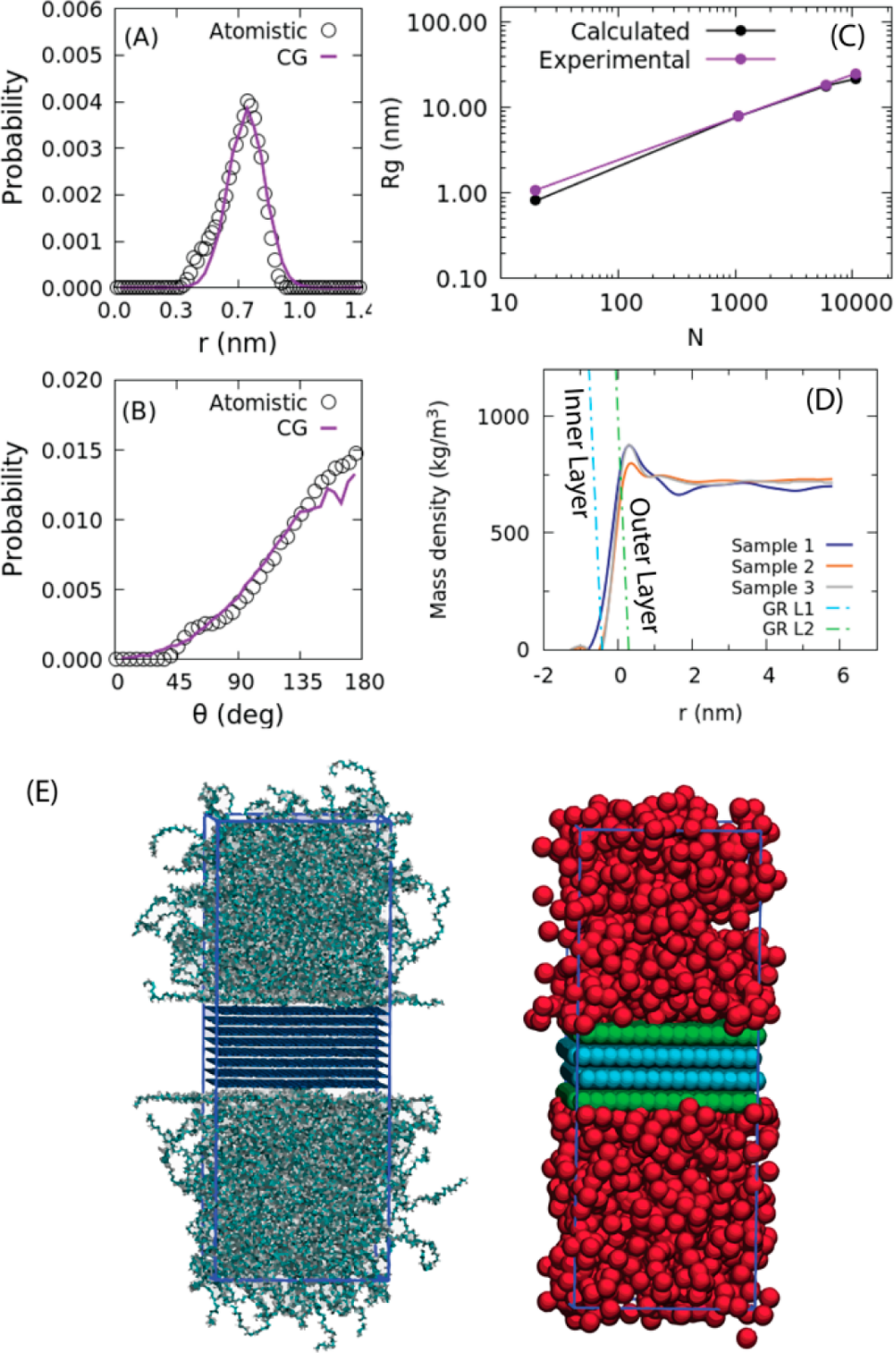
(A) Bond length and (B) angle distributions between consecutive CG beads of PE from atomistic (empty circles) and CG simulations of melts at 550 K. (C) Gyration radius of CG PE models of PE melts, as a function of chain length, compared with data from SANS experiments^62^ at *T* = 423 K. (D) Mass density profile of PE20 on the graphitic surface (GR). The position of the origin is located at 1.5 nm (i.e., half of thickness of the layers) from the center of mass of the graphitic layers. Several different χ parameter sets have been used, and two of them (sample 2, sample 3) together with the atomistic reference simulation (sample 1). The best χ parameters set (corresponding to sample 3) is the one with χ_PE,GL2_ = −4.25 kJ/mol (PE vs green layer) and χ_PE,GL1_ = 200 kJ/mol (PE vs blue layer). (E) Snapshots of the atomistic system (on the left) and hPF CG system (on the right) used for the parametrization of the χ_PE,GL1_ and χ_PE,GL2_ interaction parameters. The two external layers of the CG representation of the graphitic layers (in green) correspond to the L2 bead type, while in light blue are reported the beads of type L1.

In the Figure 3A and B, bond and angle distributions obtained from atomistic and CG models (system PE20) are compared. In Figure 3C, the behavior of the radius of gyration of CG PE chains containing up to more than 10 000 repeating units is reported (systems from PE20 to PE10696). From the figure, it is clear that the CG model gives a good reproduction of the chain size in all the explored range.

In order to estimate the acceleration of dynamics, due to coarser models and the effect of smooth potentials, a comparison between calculated and experimental diffusion coefficients is reported in the SI, section 3.2. For smaller chains in system PE1072, a scaling factor τ_CG_ ∼ 70 is obtained, while for the longer chains of system PE10796, due to the absence of entanglements in particle-field models, a larger value of τ_CG_ ∼ 700 is found. This can allow only qualitative information about the order of magnitude of this effect for each molecular weight. For a correct and quantitative description of chain dynamics, the models proposed here can be combined with the slip-spring algorithm as recently shown for CG and atomistic models of polymer melts.^60,61^

### Tuning of PE/Graphite Interaction

According to eq 2, the nonbonded interaction between PE beads and graphitic planes is tuned by the χ parameter. This parameter between PE and GR beads has been adjusted to reproduce the density profile of PE obtained from atomistic simulations (see Figure S9 in the Supporting Information). Details about the reference atomistic simulations used to parametrize the CG model of a system having eight graphitic layers in contact with a PE melt are reported in the Supporting Information, section 1.4.

In the CG models, as schematized in Figure 3E for system GR/PE20 (see Table 2 for system description), two different bead types have been considered for graphite. The layers in direct contact with the PE melt (L2 in Figure 3D, green beads in Figure 3E) have been modeled using attractive interactions with PE beads, whereas internal layers have repulsive interactions with the polymer (L1 in Figure 3D, light blue beads in Figure 3E). From Figure 3D (see also Figure S6), it is clear how this choice and the corresponding parametrization gives a good reproduction of the density profiles calculated from the reference atomistic simulation.

**Table 2:** Simulated Systems: CG Models of PE Melts in Contact with a Graphitic Surface (GR) or Primary Carbon Black Particle (CB)^a^

system	no. PE chains	total no. particles	box size (nm) [*x*, *y*, *z*]	time (μs)
GR/PE20^b^	388	3024	5.88, 6.542, 15.55	0.6
GR/PE1072	636	192128	29.43, 26.3, 59.181	0.44
GR/PE5880	116	192200	29.43, 26.3, 59.19	3.78
GR/PE10696	150	440124	35.136, 39.45, 77.19	1.95
CB/PE1072^c^	1736	495350	50, 50, 50	0.57
CB/PE5880	316	494622	50, 50, 50	1.02
CB/PE10696	174	495378	50, 50, 50	2.380

a All systems reported in this table have been simulated at 550 K. Systems are indicated as GR or CB followed by PE and the number of repeating units of the model polymer melt. The number of repeating units in one PE chain, according to the mapping scheme, is 4 times the number of coarse-grained beads.

b The GR in the system GR/PE20 is composed of four layers, each one containing 380 beads (1520 total beads). In the case of GR/PE1072 and GR/PE5880, each one of the four layers contains 5420 beads (21 680 total beads). For the system GR/PE10696, each layer contains 7715 beads (30 860 total beads).

c The CB is formed by a core and a shell. The core contains 796 molecules, each one composed of four beads (3184 beads in the core). The shell contains 626 molecules, each one composed of 43 beads (26 918 beads in the shell). In total, a CB primary particle counts for 30 102 beads.

### CB Primary Particle

**Figure 4:**
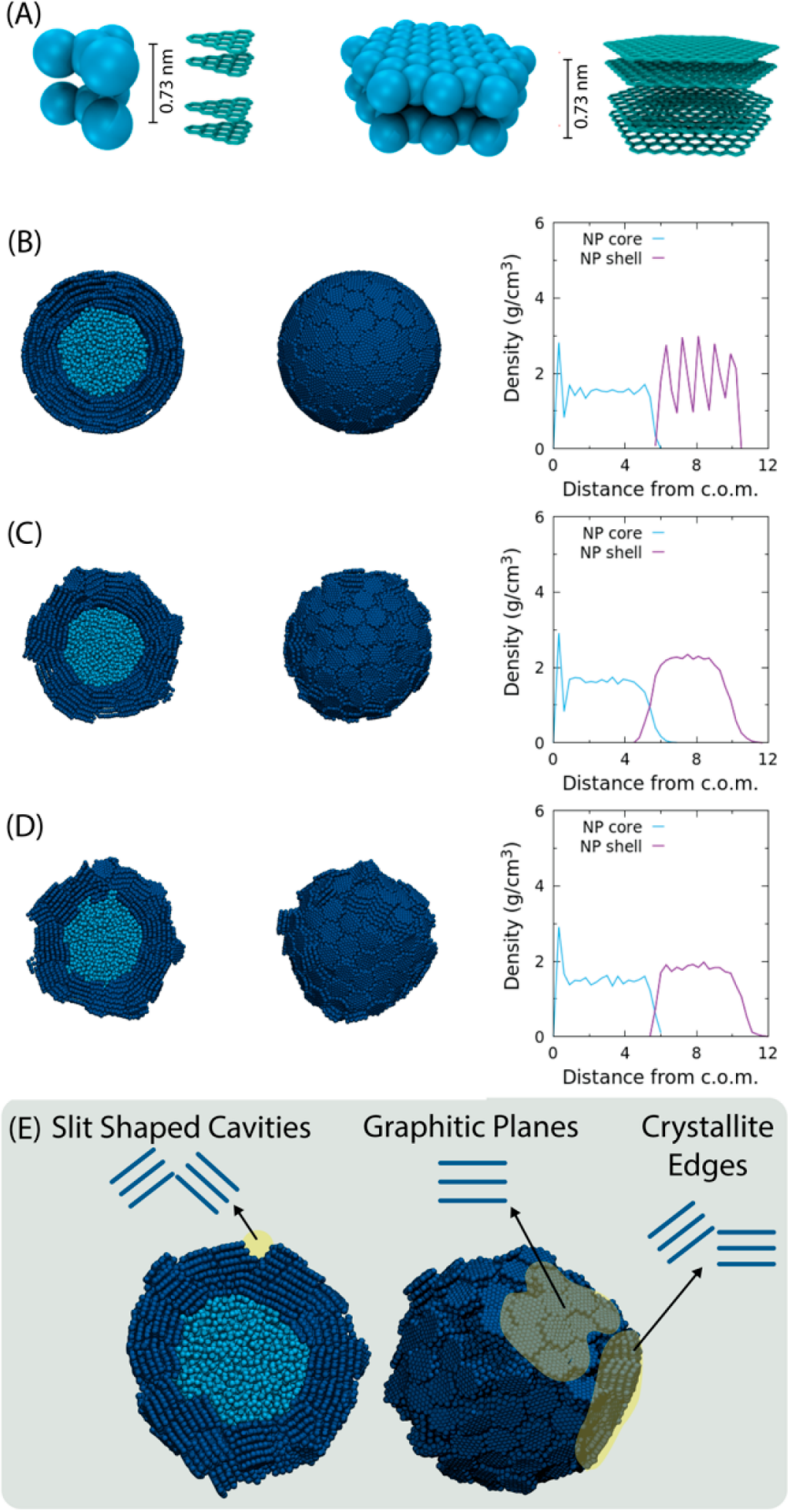
(A) Atomistic and CG structures of pairs of core (right) and shell (left) units. Density profiles corresponding to different stages of the procedure to obtain CB primary particles are reported. (B) Initial configuration of core (light blue) and shell (blue) molecule arrangements (system CB). (C) Structure of system CB obtained after first annealing stage. (D) Final configuration of system CB. (E) Two views of the final configuration of system CB showing different sites on the CB surface. Planar graphitic surfaces (type I site), crystallite edges (type III site), and slit shaped cavities (type IV site) are highlighted in the picture.

The CG model of a primary particle of CB has been set up according to previous several experimental and modeling literature results. According to microscopy and X-ray diffraction observations, the surface of primary particles is made of graphite layers arranged in a concentric fashion with significant distortion. Several experimental studies indicate models of carbon blacks with a tendency toward the concentric alignment of graphite layers roughly parallel to the external surface of carbon particles.^63−65^ On the basis of these studies, an atomistic model of a primary particle of CB has been proposed recently by Ban et al.^66^ This atomistic model consists of a core–shell structure in which two regions are distinguished as the amorphous core and the graphitized shell. Inside the amorphous core, smaller graphite sheets are included, while for the external graphitized shell, larger units are considered. In Figure 4A, the atomistic structure and the resulting CG models for the two different core and shell units are described. In particular, the hexagonal shell units, constituting the shell of the CB particle, have a length of 2.95 nm similar to those (2.7–3.7 nm) of the atomistic model reported by Ban et al.^66^ Interactions among CG beads modeling the different units present in the CB primary particles, are calculated through Lennard-Jones pair potentials. This choice is a necessary ingredient to ensure the so-called “paracrystalline behavior” of CB particles with layers concentrically arranged around a growth center.^1^ Details about both bonded and nonbonded parameters used herein and atomistic reference models of core and shell molecules can be found in the Supporting Information.

In Figure 4B–D, snapshots corresponding to the different stages of the procedure adopted to construct a primary particle CG model of a diameter of 20 nm are reported. The core–shell structure is obtained by first arranging the smaller graphitic units inside a sphere of 10 nm diameter. After minimization and constrained MD simulations of the inner core, a layer of 10 nm thickness is obtained by packing shell units stacked in concentric layers in which the graphitic surfaces are tangent to the surface of a sphere having a diameter of 20 nm. The composition of the primary particle model (system CB) is described in Table 2 and Table S14 of the SI. As can be observed from Figure 4B, in the initial structure of the CB particle, the surface is very regular. Starting from this configuration, a first annealing procedure in which the inner core has been restrained was executed. A snapshot of the final structure at this stage is reported in Figure 4C. Starting from this configuration, a second set of annealed MD simulations in which both core and shell united have unrestrained motion has been performed. Complete information about the initial structure setup and the annealing procedures are reported in the Supporting Information. From the pictures and the plots of Figure 4B–D, it can be observed that starting from the regular initial structure, where shell molecules are placed around the core in an ordered way, during the equilibration, a more disordered structure is developed at the surface of the CB particle, where the density is smoother throughout the whole external shell. The mean density, resulting from the contribution of the inner core and the outer graphitized shell, is around 2 g/cm^3^, which agrees well with the experimental measurements (2.05–2.25 g/cm^3^).^24^ Interestingly, from visual inspection of the configurations, it is possible to observe the development of different structure sites obtained by piling up three or more shell units. A para-crystalline structure of the CB primary particle is obtained as a spherical arrangement of several ordered layered units, defining different organized regions (microcrystallites) with defects at their boundaries. In particular, as highlighted in Figure 4E, on the particle surface, together with regular planar surfaces involving several adjacent shell molecules, it is possible to observe two different surface sites such as crystallite edges and slit shaped cavities. These arrangements are important features of the proposed model since, from an experimental point of view, they are considered important adsorption sites for polymer chains.^14,23,29,30,67^ In particular, four discrete species (named I to IV) irrespective of morphology have been identified by analyzing surface energy distribution of CB obtained from static gas adsorption experiments.^38^ Type I adsorption sites are indicated as planar graphitic surface regions having sp^2^ hybridization. Sites of type II correspond to amorphous. Sites III and IV are attributed to the edges of microcrystallites and to slit shaped pores, respectively. These different surface sites and their relative abundancies are related to the surface disorder of the microcrystallites present on the surface of CB primary particles.

**Figure 5:**
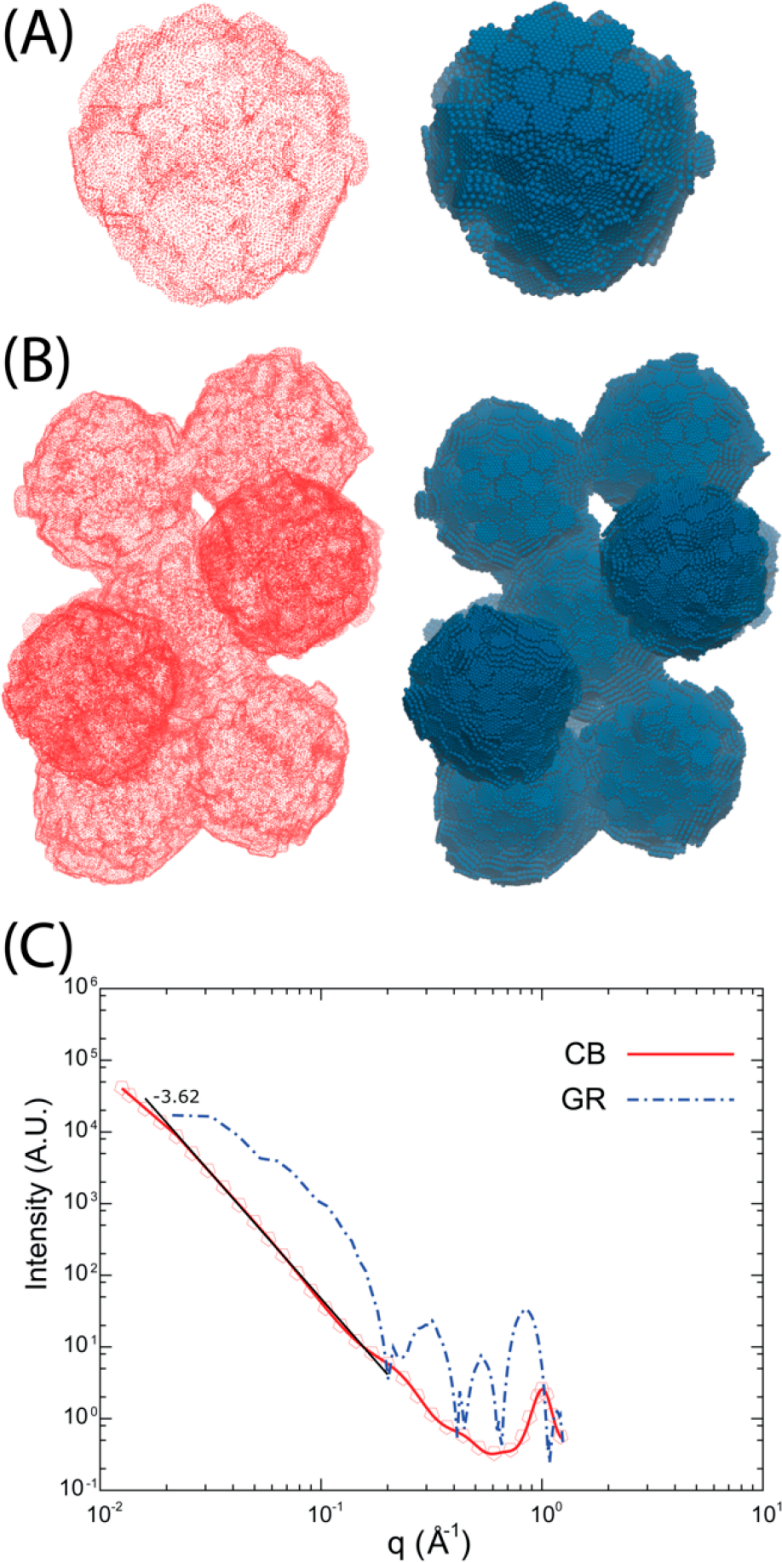
Solvent accessible surfaces area (SASA; in red) and the corresponding configuration (on the right) used to calculate it reported for a single primary CB particle (A) and for an aggregate counting nine CBs (B). The probe radius used to calculate the SASA is 0.365 nm. (C) SAXS profiles calculated for the GR (system GR) and CB primary particle (Table S14 of SI). The behavior of the scattered intensity fitted as power law decay for the CB model is also reported as a black line.

Another possible way to quantify and to compare the surface structure obtained from the proposed CG models with the experiments is the accessible surface area (SASA), which has been calculated by using the configurations reported in Figure 4.^68^ (see Figure 5A) The more compact and regular initial structure of Figure 4B has the smallest surface area (1530 nm^2^), while the final structure of Figure 4D has a larger area (1760 nm^2^). The experimental value of the surface area of CB depends on the production and processing conditions and for finely dispersed CB aggregates is ∼200 m^2^/g. This value, considering the total mass of the proposed core–shell model, would correspond to a contribution of ∼1600 nm^2^ per particle, which is not far from the value calculated from the model of one single primary particle. However, the experimental related value should be considered as a lower bound. Indeed, even in a finely dispersed state, CB is never present as single primary particle, and the smallest dispersible units have been evaluated to correspond on average at least to ∼9–18 particles. For this reason, to improve the estimation, we simulated the aggregation process, using as a starting configuration nine primary particles at the final stage of the procedure (structures corresponding to Figure 4D), to obtain the structure of an aggregate (more information about the MD simulation of the aggregation process is provided in the Supporting Information). The aggregate structure (together with a visualization of the SASA) is reported in Figure 5B. The SASA calculated for the nine primary particles’ aggregate is 15 615 nm^2^: this value corresponds to a contribution of 1735 nm^2^ per particle that is closer to the experimental value of ∼1600 nm^2^ per particle.

A further validation of the proposed CB model can be obtained by calculation of X-ray scattering from MD simulations. Indeed, small-angle X-ray scattering (SAXS) studies have been widely employed to characterize surface roughness in CB as a powder or dispersed in polymers.^69−71^ The measured scattering patterns for CB are characterized by a surface-fractal-like power-law decay of the intensity as a function of scattering vector *q*. This decay is described in terms of surface-fractal models, related to particles with fractal rough surfaces. In a range of *q* going from the size of a primary particle (typically *q*∼ 0.01 Å^–1^) to the scale of few graphitic units (*q* ∼ 1.0 Å^–1^), the scattering intensity follows a power low decay of the type *I*∝ *q*^*D*_s_–6^, where *D*_s_ is the fractal dimension of the CB surface. For a regular two-dimensional surface, *D*_s_ = 2, and the intensity decays as *q*^–4^.^69^ For rough fractal surfaces (*D*_s_ > 2), the decay is expected to be slower as described by Bale and Schmidt.^72−75^ Typical exponents obtained for CB fillers are in the range of −3.7 to −3.4 depending on filler type and its concentration.^69−71^ In Figure 5C, the SAXS intensity calculated from an MD trajectory of 100 ns of system CB at 550 K is reported as a function of *q*. The data, as reported in Figure 5C, are well described by a decay law fitted as ∝*q*^–3.62^ in good agreement with the typical experimental values obtained for CB samples. This result is a further validation of the proposed model of a CB primary particle, in particular, a validation of the disorder of the shell molecules on the particle surface. In the same Figure 5C, for comparison, is also reported the behavior of scattered intensity calculated from the regular graphitic surface model. From the plot corresponding to the regular surface of system GR, it is clear that the behavior is not only quantitatively but also qualitatively different from the experimental features of CB samples. In particular, the intensity does not follow a power law, and the presence of regularly stacked flat planes and, in the same plane, regularly spaced beads give rise to the obtained pattern.

### Mesoscale Simulations of CB/PE Interfaces

**Figure 6:**
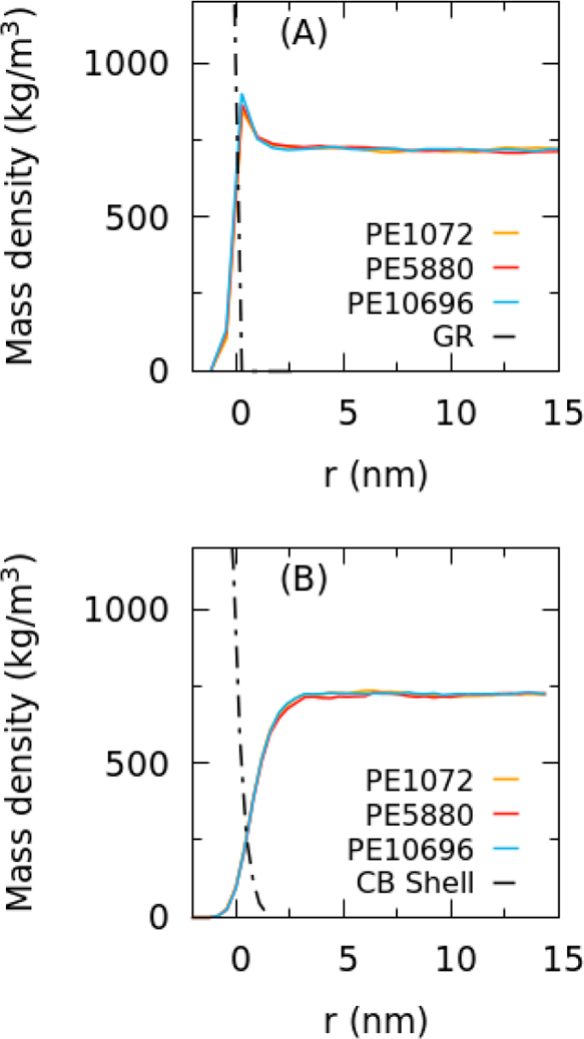
Density profiles for (A) GR and (B) CB surfaces for PE systems having different chain lengths. The *x* axis indicates, for both systems, the distance from the surface. The position of the origin for the CB particle is located at 10 nm (i.e., the radius of the CB particle) from the center of mass of the particle. For the GR, the position of the surface is located at 1.5 nm (i.e., half of thickness of the layers) from the center of mass of the graphitic layers.

As a first application of the proposed model, large scale simulations of systems having monodisperse PE melts of different chain lengths have been performed. Due to the large sizes of the CB particle diameter (20 nm) and of the chain lengths of PE, in order to avoid finite size effects, the box length of simulated systems has been set to 50 nm (2.5 times larger than CB diameter). This corresponds to systems of ∼500 000 beads. The list of simulated systems described in the present section is in Table 2. Systems are indicated as GR or CB followed by chain lengths (expressed as number of PE monomers equal to 4 times the number of CG beads). For both GR and CB, for the systems reported in this section, beads corresponding to the filler models are kept fixed by excluding them from the MD integration algorithm. For both GR and CB model fillers, as also explained in the previous sections, beads present on the external surface experience attractive interactions with the PE; on the contrary, internal beads have repulsive interactions with PE (model parameters are reported in Figure 3 and in section 2.5 of SI). To compare and to understand the effect of CB particle curvature and surface disorder, for each system modeling a different CB/PE interphase, an analogous system having flat and infinite graphitic layers (GR) has been simulated. The density profiles for GR and CB surfaces for PE systems having different chain lengths (systems GR/PE1072, GR/PE5880, and GR/PE10696 and CB/PE1072, CB/PE5880, and CB/PE10696) are reported in Figure 6: it is apparent that the behavior of density profiles in each GR and CB system is very similar for different chain lengths. Instead, the behavior of PE close to GR and CB surfaces is different. In particular, the PE density at the GR surface is very similar to the one calculated from atomistic simulations showing higher density close to the surface. On the contrary, for CB systems, the density grows smoothly from zero to the bulk value without any peak. These different behaviors are related to the different structures of the GR and CB surface, and the latter, as previously discussed, is characterized by surface disorder. Indeed, the density profile of the CB surface shows a smoother behavior, large surface area, and large interpenetration between PE and the solid interface. Interpenetration lengths (IL) have been calculated from density profiles (see eq S6) and reported in Table S16. For the flat surface IL (calculated for all considered systems) is ∼2 nm, while larger values (∼2.8 nm) have been obtained for CB systems. This different behavior between GR and CB surfaces can be qualitatively compared with experimental data about the CB/*cis*-polybutadiene interphase in which larger bound layers have been found for fillers with higher surface areas.^76^ More subtle differences have been found for the IL as a function of molecular weight of PE. In particular, for both CB/PE1072 and CB/PE5880, the calculated IL is ∼2.8 nm, while for CB/PE10696 it is 1.98 nm. Differently, when going from GR/PE1072 to GR/PE5800, an increase of IL from 2.0 to 2.38 nm is obtained, while for the largest chain length GR/PE10696, a value of 2.24 nm is found.

The CB/PE interphase can be further characterized by calculating the work of adhesion *W*_ad_ between the PE melt and a single CB primary particle. In our approach, the average value of the work of adhesion ⟨*W*_ad_⟩ can be calculated as^51,77^
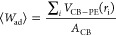
2where *A*_CB_ is the surface area of the primary CB particle calculated as SASA, as discussed in the previous subsection, and *V*_CB-PE_ corresponds to the first term of the right-hand side of eq 1 summed over all beads *i* constituting the CB particles and the PE melt. For all the considered systems, the calculated ⟨*W*_ad_⟩ is 124–128 mJ/m^2^, in fair agreement with the experimental value of 102 mJ/m^2^ available for HDPE at 450 K.^78^

**Figure 7:**
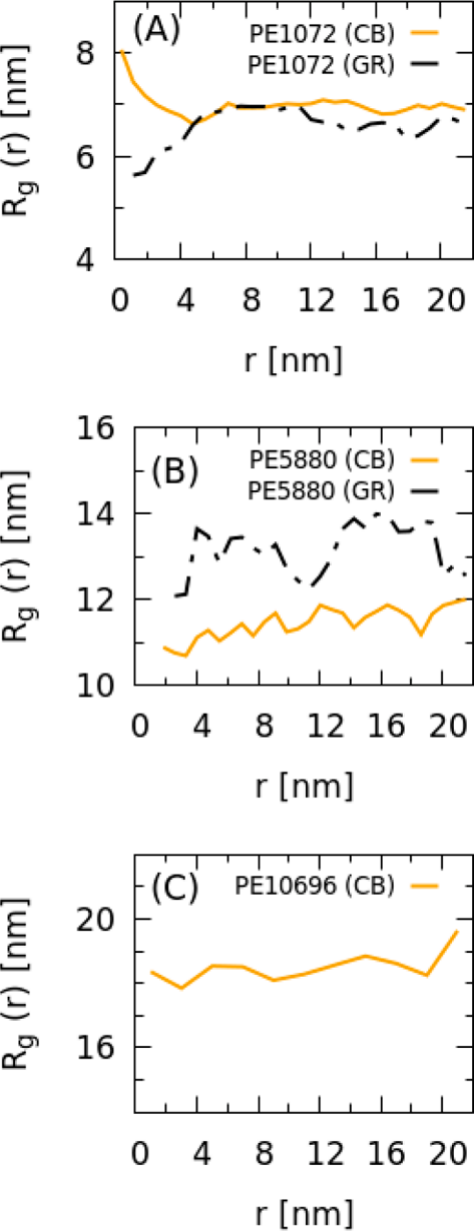
Gyration radius (Rg) of PE chains as a function of the distance from the solid surface calculated for the systems. (A) GR/PE1072 and CB/PE1072, (B) GR/PE5880 and CB/PE5800, and (C) CB/PE10696.

Different behaviors between GR and CB surfaces are obtained also for size and conformations of PE chains in contact with the surfaces. In particular, for CB, a tendency to increase the chain size at a short distance from the surface is obtained, while the opposite tendency is observed for GR (see Figure 7). These results are consistent with previous simulation studies about spherical nanoparticles in contact with polymer chains^47,50,79,80^ and for the flat GR surface with the results of Harmandaris et al.^81,82^

**Figure 8:**
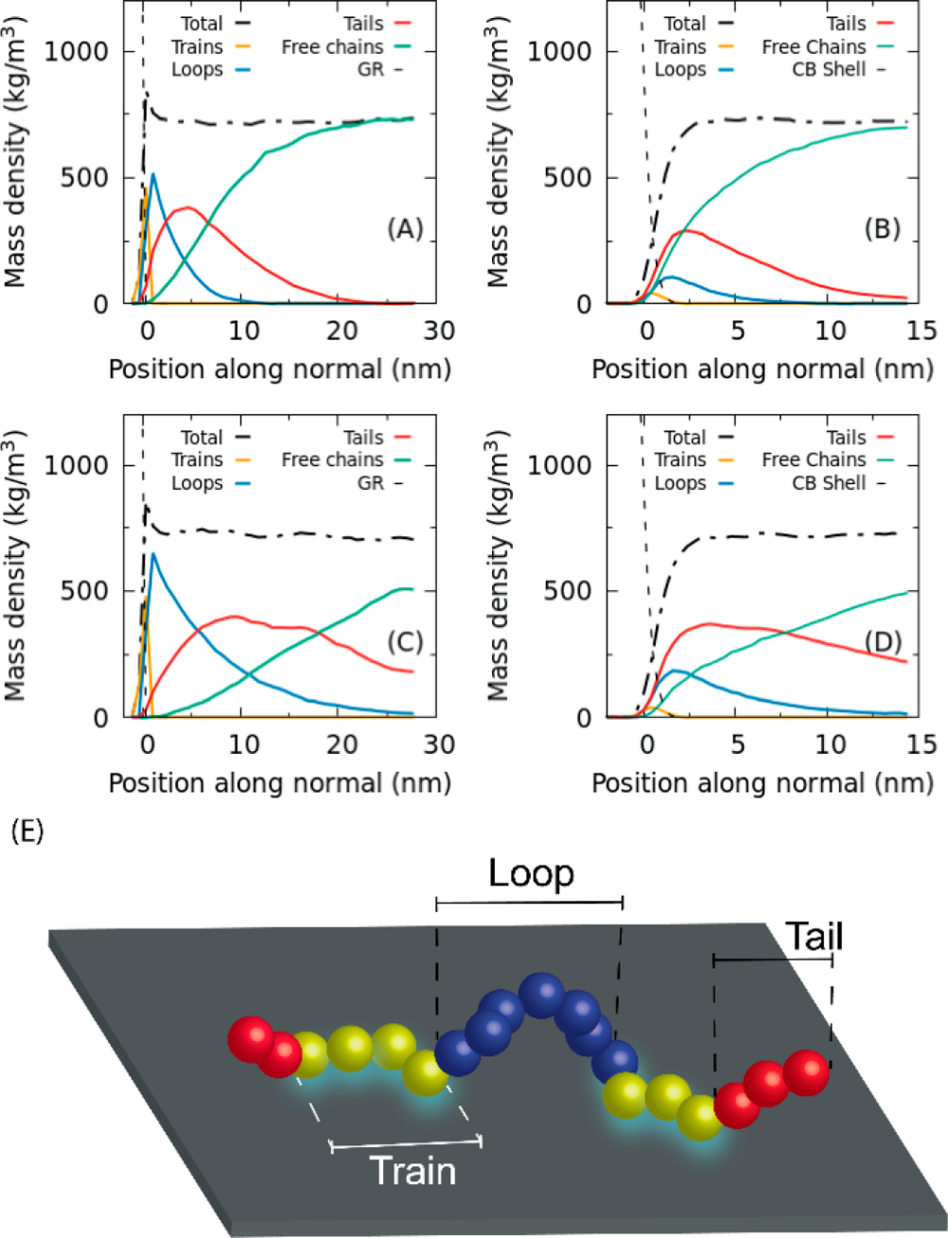
Behavior of partial density profiles of trains, loops, trains, and free chains for PE chains as a function of distance for system (A) GR/PE1072, (B) CB/PE1072, (C) GR/PE5880, and (D) CB/PE5880. (E) Sketch of a typical conformation of an adsorbed chain.

To gain a detailed picture of adsorbed PE on the GR and CB surfaces, statistics on conformations formed by chains adsorbed on interfacial area have been obtained analyzing trajectories of MD simulations. Different possible conformations of adsorbed chains, namely, trains, loops, and tails, have been considered.^82,83^ At the bottom of Figure 8, a scheme sketching a typical conformation of an adsorbed chain is depicted. In particular, trains are defined as successive beads within a distance inside the adsorbed layer (a cutoff distance ≤ 0.73 nm from the GR or CB surface has been considered). Loops are sequences of beads connecting two trains outside the adsorbed layer, and finally, tails are sequences of beads connected only from one side to a train and protruding toward the bulk polymer phase.

**Figure 9:**
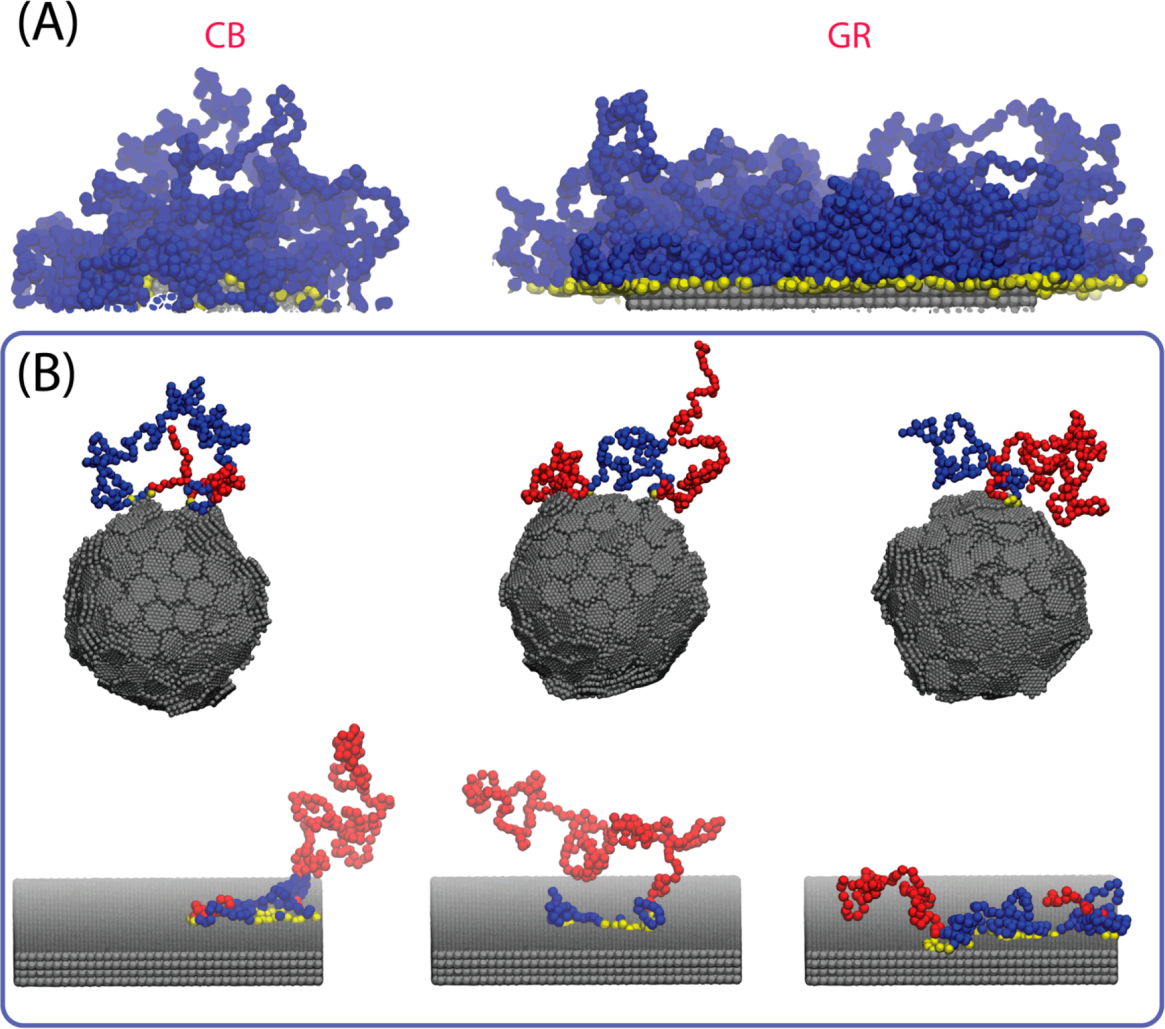
(A) Snapshots showing conformations of trains (yellow beads) and loops (blue beads) adsorbed on CB and GR surfaces (systems CB/PE1072 and GR/PE1072). In both cases, the beads of CB and GR are reported in gray. (B) Typical configurations of adsorbed PE chains on the CB surface (on top of the panel system CB/PE5880) and GR plane (system GR/PE5880) on the bottom of the panel). Train segments (yellow), loops (blue), and tails (red) are colored differently to guide the reader.

In Figure 8A–D, partial density profiles calculated from beads belonging to trains, loops, tails, and free chains as a function of distance from the CB and GR surfaces are reported. From these figures, it is clear that for all systems the quantity of free chains approaches a plateau at a distance from the surface of ∼2Rg, and accordingly, at the same distance, the concentration of all types of adsorbed chains (trains, loop, and tails) is zero. This distance can be considered as a thickness of the bound layer; similar results have been reported from atomistic simulations of Müller–Plathe and co-workers.^36,37,80^ As for adsorbed chains, the main difference between the two surfaces is that, in the case of the flat GR surfaces, trains (red curves) and loops (light blue) show higher concentrations close to the surface (up to ∼2 nm from the surface), while tails are more abundant at an intermediate distance between 2 nm and 2 Rg. Conversely, in the presence of the CB particle, the loop concentration is always smaller with respect to trains, tails, and free chains. This behavior is consistent with the effects observed on the chain size of the CB and GR surface. In particular, chains more compressed along the direction perpendicular to the interface can be observed for the flat GR surface with loops that are shorter and closer to the surface. On the contrary, chains more loosely bound to the CB surface have less localized and more open loops and a large concentration of tails. Both behaviors are consistent with the increase of size observed for chains close to the CB (increase of size) and GR (decrease of size) surfaces. The different structural features of PE chains adsorbed on CB and GR surfaces are very clear in the snapshots depicted in Figure 9A and B. From these snapshots, it is clear how the loops (blue beads) in the case of planar GR surfaces are closer to the surface while the loops in the case of the CB primary particle show a more open structure.

**Figure 10:**
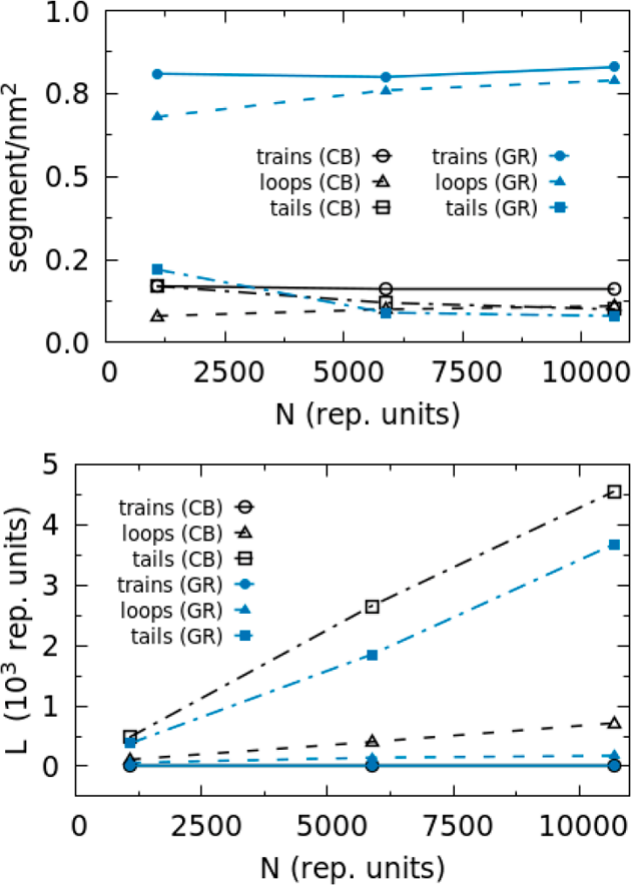
Top panel, number of segments/nm^2^, where segment can be train, tail, or loop, calculated for systems having both CB and GR surfaces. In the bottom panel, the length of segments is calculated and reported for the same systems.

To gain a more detailed picture of the conformational behavior of adsorbed chains, simulations of different systems have been analyzed in terms of total number of trains, loops, and tails. In order to compare systems of various sizes, the number of different chain conformations has been normalized by the contact area; according to this prescription, henceforth the number of trains, loops, and tails per square nanometer will be reported. According to the data already shown in Figure 8, the number of adsorbed conformations per unit area on the CB primary particle is smaller with respect to the flat GR surface (see Figure 10 and Table 3). This is simply related to different PE density in contact with the two different surfaces. In both cases, the number of trains does not change with PE chain length, and a similar behavior is observed for the train length. Long trains of 11–12 monomers are formed on the GR surface: these data are in agreement with atomistic simulations of similar systems.^81,82^ Due to the surface disorder, shorter sequences of monomers (about six) are involved in a trains arrangements at the CB surface. A slight increase of the number of loops and a decrease of the number of tails are observed as a function of the chain length for both CB and GR surfaces. At the CB surface, trains and tails are more abundant than loops, while at the flat GR surface the numbers of trains and loops are larger than that of tails. As for the sequence lengths, loops formed at the CB surface are always longer (about 100 and 400 repeating units for systems CB/PE1076 and CB/PE5880, respectively) than ones formed at the GR surface by PE chains of the same length (about 60 and 140 for systems GR/PE1076 and GR/PE5880, respectively). On the contrary, sequences of repeating units forming tails at CB and GR surfaces show similar lengths.

**Table 3:** Conformational Statistics for Adsorbed PE Chains^a^

system	trains/nm^2^	loops/nm^2^	tails/nm^2^	trains length	loops length	tails length
GR/PE1072	0.81	0.68	0.22	11.0	58.0	376
GR/PE5880	0.80	0.76	0.09	11.6	143	1852
GR/PE10696	0.83	0.79	0.08	12.0	176	3680
CB/PE1072	0.17	0.08	0.17	6.0	112	480
CB/PE5880	0.16	0.10	0.12	5.9	404	2656
CB/PE10696	0.16	0.11	0.10	6.0	716	4555

a The segment length (train, loop, and tails) is reported as repeating units.

### Large Scale Simulations Benchmarks

**Figure 11:**
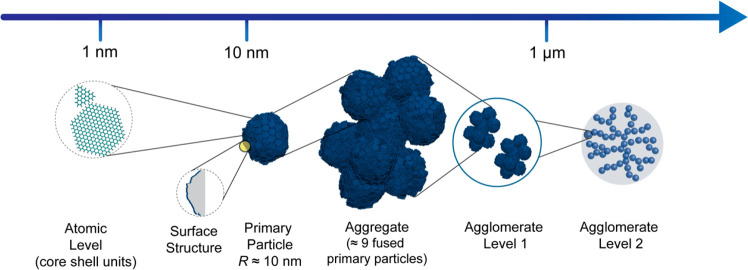
Hierarchical structure of CB ranging from the subnanometer (atomic scale of core and shell units) approaching the micrometer scale for the agglomerate structures involving about 20 primary particles.

If the molecular models here introduced would be efficiently employed on large scale systems involving multiprimary particles aggregates (or even agglomerates), they may open the possibility of a computational screening involving the optimal combination of surface chemistry modifications, size and shape of CB aggregates, and the chemical structure of the polymer, achieving an important tool to address the polymer–fillers interface and interphase engineering in the polymer industry. Such an approach must be pursued using a molecular model on a scale taking into account the hierarchical nature of the filler, i.e., spanning from the molecular scale up to the typical length-scale of CB dispersible units. As sketched in Figure 11, such units are in a range between a single CB aggregate (clusters consisting of about nine CB primary particles) and a CB agglomerate (consisting of units between one or two aggregate, about 9–18 CB primary particles).

**Figure 12:**
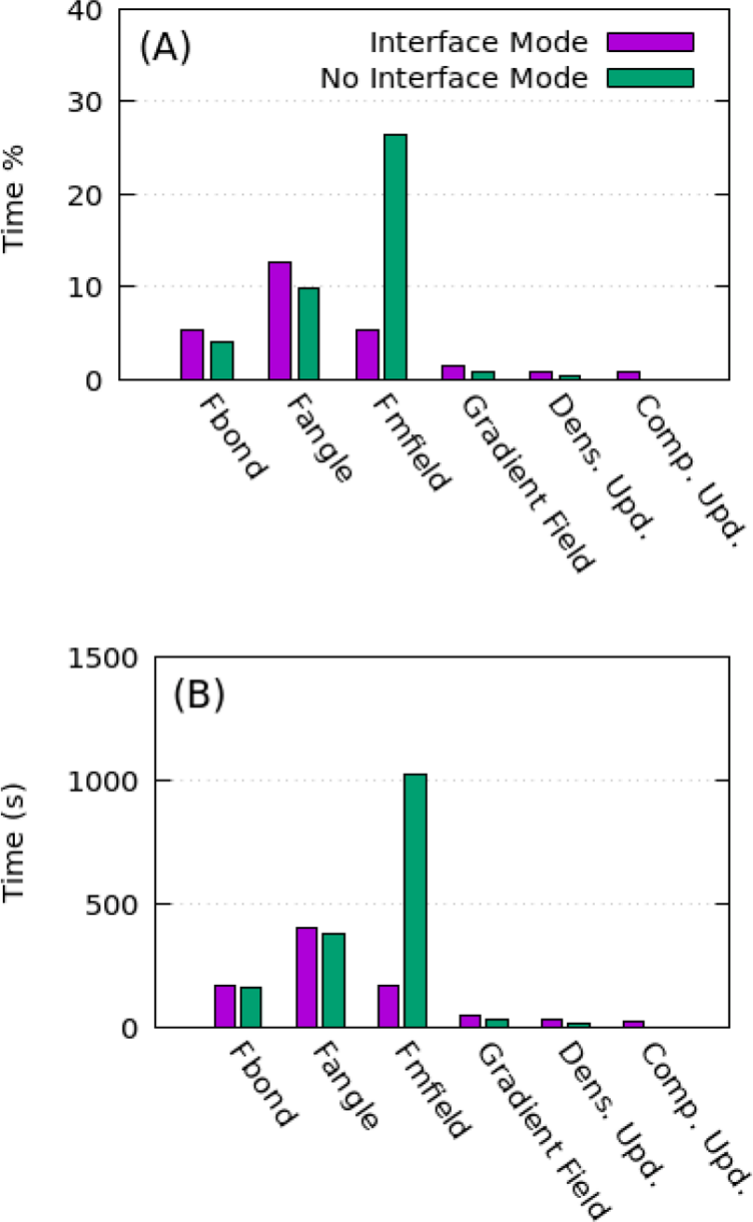
Comparison of profiles using Scalasca 2.4 profiler.^84^ The profiles show: (A) percentage of time spent by subroutines and (B) absolute time. The system used to calculate the profiles is composed of 1 NP (see Table 4). The Δ*t*_update_ was set equal to 3 ps. When used, the IM update was set equal to 3 ps. The simulations (10^5^ steps) have been performed on 48 cores.

For the systems considered in this paper, code profiles (obtained from Scalasca 2.4 profiler^84^) are reported in Figure 12. In particular, panel A shows that the evaluation of forces due to the density fields (see Fmfield bar) takes about 25% of the total computation time. From Figure 12, it is clear how the cost of density and density gradient interpolation is largely reduced for the simulation using IM, while the cost of a composition update is quite small.

To compare the efficiency of the optimized version of OCCAM, systems containing from 1 to 18 CB particles have been simulated for 100 000 time steps. The Δ*t*_update_ has set to 3 ps, like the value used in production runs. When it is used, the interface mode (IM) is updated every 3 ps with the same frequency of density field updates. In Table 4, system composition and performances are listed for both versions of the OCCAM code and for runs using the IM.

**Table 4:** Benchmarks Performed on Systems Containing 1, 2, 9, and 18 Carbon Black Primary Particles within PE Polymer Melt, with 10% w/w of Carbon Black^a^

				performances (10^6^ steps/day)		
systems	no. particles	no. core	average particles/core	standard OCCAM^58^	S3	S3 (IM)^b^
CB/PE1072	460.560	48	9595	7.7	22	32.6
		96	4797	8.6	30.2	53
CB/PE1072	932.370	96	9712	3.1	22.9	37.7
		192	4856	3.2	62.6	80
9-CB/PE1072 (aggregate)	4.196.466	432	9714	0.6	12.1	16.3
18-CB/PE1072 (agglomerate)	8.392.932	864	9714	0.3	0.76	1.26

a All simulations have been performed on an Intel Xeon E5-2697v4, 2.30 GHz, 18 Core/CPU with Infiniband QDR/True Scale Broadwell.

b The (IM) has been used. The IM update frequency is set equal to Δ*t*_update_ (3 ps).

**Figure 13:**
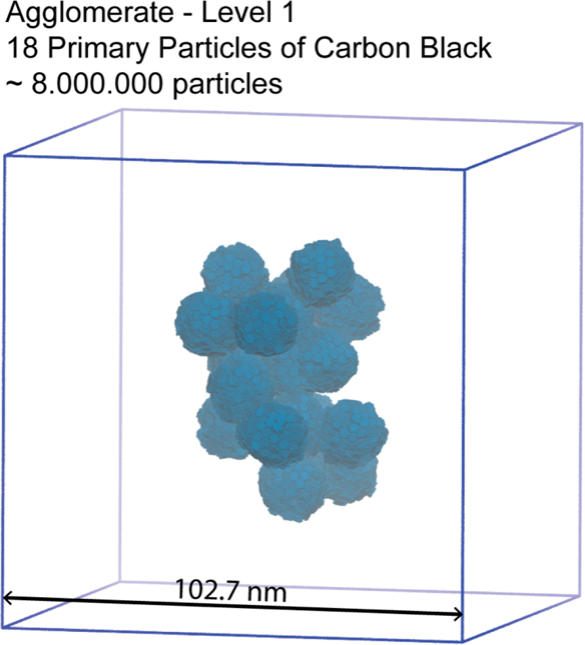
Snapshots of system 18-CB/PE1072 containing an agglomerate of CB in PE bulk. The agglomerate of level 1 is composed of 18 CB primary particles (in agreement with the experimental average number of CB particles). The side length of the cubic box is 102.7 nm. For clarity the PE polymer chains are omitted. The composition of the system is reported in Table 4.

The optimized code has performances, depending on the system size, ranging from a speed up of ∼3 to ∼20 times with respect to the standard code. The increase of performances due to IM gives a speed of up to 1.3–1.8. Benchmarks, reported in Table 4, for systems ranging from one to 18 CB primary particles embedded in PE melts (total number of beads ranging from ∼500 000 to more than 8 000 000) indicate that long MD simulations are feasible, employing a moderate number of cores (ranging from ∼100 to 900). For example, for the two largest systems embedding nine and 18 primary particles (a snapshot of the system counting 18 primary particles is shown in Figure 13), production runs of about 16 and 1.3 million of steps can be obtained in 24 h.

## Conclusions and Perspectives

In the present study, an efficient coarse-grained model suitable for CB/PE interfaces and interphases is proposed. The model, based on reference atomistic simulations, is described and validated. Large-scale simulations have been performed considering a realistic size of a CB particle diameter (20 nm). The behavior of adsorbed chains is different when they are in contact with an infinite graphitic surface and more realistic CB surfaces having surface disorder and correct size and curvature. The computational efficiency of the proposed model allows a straightforward extension of the range of application to multiprimary CB particle systems. Interfaces and interphases between aggregates (∼10 primary particles), agglomerates (level 1–20 primary particles), and PE melts can be modeled. Besides the hierarchical structure of a single primary particle showing surface disorder and realistic adsorption sites, further cavities and hindered spaces on a scale larger than the CB surface structure are expected to have an interplay between CB and polymer structures on the scale of larger chain sizes. In this way, using molecular models, a full description of the CB/PE interphase on the scale of dispersible units is achievable. Given the large increase in the development of materials informatics for the characterization and discovery of new carbon based materials,^40^ high-throughput classical MD computations, based on the proposed models, can be planned to construct data sets for machine learning algorithms. Such an approach opens the possibility of a computational screening involving the optimal combination of surface chemistry modifications and size and shape of CB aggregates achieving an important tool to address the polymer/fillers interface and interphase engineering in the polymer industry.

## Supporting Information Available

Atomistic models and simulations, coarse-grained models, and additional analysis (PDF)
