# Supplementary material for: Efficient Hybrid Particle-Field Coarse-Grained Model of Polymer Filler Interactions: Multiscale Hierarchical Structure of Carbon Black Particles in Contact with Polyethylene
Source: J Chem Theory Comput. 2021 Feb 12;17(3):1755–70. doi: 10.1021/acs.jctc.0c01095 (PMC8023654; doi:10.1021/acs.jctc.0c01095)
Supplement: Supplementary file 1 — ct0c01095_si_001.pdf [file ct0c01095_si_001.pdf]

Supporting Information

Efficient Hybrid Particle-Field Coarse-Grained Model of Polymer Filler Interactions: Multiscale Hierarchical Structure of Carbon Black Particles in Contact with Polyethylene

Stefano Caputo,[1] Velichko Hristov,[2] Antonio De Nicola,[3] Harald Herbst,[2] Antonio Pizzirusso,[1] Greta Donati,[1] Gianmarco Munaò,[4] Alexandra Romina Albunia,[2] and Giuseppe Milano*[1,3]*

[1]Dipartimento di Chimica e Biologia, Università di Salerno, Via Giovanni Paolo II, 132, I-84084, Fisciano, SA, Italy.

[2]Innovation & Technology, Borealis Polyolefine GmbH, St.-Peter-Straße 25, 4021, Linz, Austria

[3]Department of Organic Materials Science, Yamagata University, 4-3-16 Jonan Yonezawa, Yamagata-ken 992-8510, Japan

[4]Dipartimento di Scienze Matematiche e Informatiche, Scienze Fisiche e Scienze della Terra, Università degli Studi di Messina, Viale F. Stagno d'Alcontres 31, 98166 Messina, Italy

Table of Contents

1. Atomistic Models and Simulations *3*

1.1 Model of PE 3

1.2 Atomistic Model of Graphitic planes and CB’s Core and Shell Particles 5

1.3 Equilibration procedure for the atomistic systems 7

1.4 Atomistic Simulation of the GR/PE System 8

1.5 Computational Details for Atomistic Simulations 8

2. Coarse-Grained Models *10*

2.1 Model of Core and Shell CG molecules of Carbon Black (CB) 10

2.2 Annealing Procedure to build the CB primary particle 12

2.3 Aggregation of 9 and 18 CB primary particles 13

2.4 Graphitic Planes (GR) 14

2.5 χ (hPF) Parameterization 15

2.6 PE Model 16

3 Additional Analysis *18*

3.1 Interpenetration Length 18

3.2 Comparison of Diffusion coefficients 18

3.3 End to end autocorrelation functions (ACF) 19

3.4 Effect of Interface Mode on CB/PE Density Profile. 20

References *21*

1. Atomistic Models and Simulations

1.1 Model of PE

The L-OPLS force-field,[1–3] which is a version of the OPLS-AA force field optimized specifically for long chain hydrocarbons, is used for the PE polymer chains. In Figure S1, the chemical structure of PE showing the atom types and atom charges assignment, is reported. The terminal carbon atoms and their bonded hydrogens are labelled as C_T and H_T, while backbone carbon atoms and their bonded hydrogens are named C_2 and H_2. According to the OPLS-AA force-field, the non-bonded interactions are composed of two terms (eq. S1), the Lennard-Jones (LJ) term (left side of the right hand of eq. S1) and the Coulombic term for the electrostatic. The 1-4 intramolecular nonbonded interactions are scaled down by a factor f_{ij} = 0.5, otherwise f_{ij} = 1. In Table S1, the parameters for the non-bonded interactions are listed.

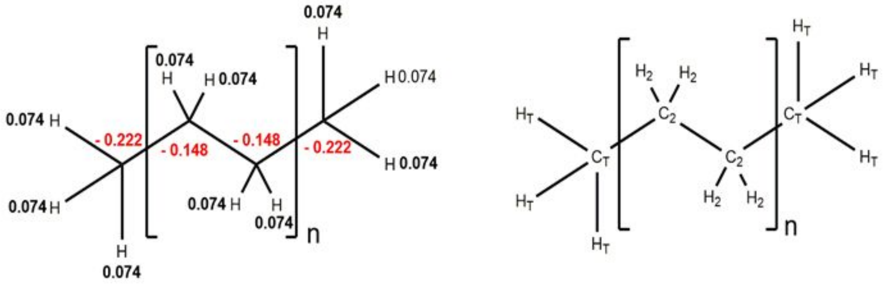

Figure S1. Polyethylene (PE) monomer structure with partial charges scheme (left) and atom types (right).

$$V(r_{ij}) = 4\varepsilon_{ij}\left[\left(\frac{\sigma_{ij}}{r_{ij}}\right)^{12} - \left(\frac{\sigma_{ij}}{r_{ij}}\right)^{6}\right] + \left(\frac{1}{4}\pi\varepsilon_0\right)\left(\frac{q_i q_j}{r_{ij}}\right) \quad \text{(S1)}$$

Table S1. Non-bonded interaction parameters of PE.

Atom Types	σ (Å)	ε (kJ mol^{-1})	q
C_T	0.350	0.276144	-0.222
C_2	0.350	0.276144	-0.148
H_T	0.250	0.125520	0.074
H_2	0.250	0.110000	0.074

The functional form for bonded interactions, including bonds, angles, and torsions, are reported in the following.

$$V_b(\mathbf{r}_{ij}) = \frac{1}{2}K_{bond}(\mathbf{r}_{ij} - \mathbf{r}_{ij}^0)^2 \qquad \text{(S2)}$$

$$V_a(\theta_{ijk}) = \frac{1}{2}K_{angle}(\theta_{ijk} - \theta_0)^2 \qquad \text{(S3)}$$

$$V_d(\theta_{ijkl}) = \frac{1}{2}[C_1(1 + \cos\phi) + C_2(1 - \cos 2\phi) + C_3(1 + \cos 3\phi) + C_4(1 - \cos 4\phi)] \quad \text{(S4)}$$

$$V_{rb}(\theta_{ijkl}) = \sum_{n=0}^{5} C_n(\cos(\psi))^n \qquad \text{(S5)}$$

Table S2. Harmonic bond potential (Eq. S2)

**Bonds**	$\boldsymbol{K_{bond}}$ (kJ mol^{-1} Å^{-2})	$\boldsymbol{r_{ij}^0}$ (Å)
C_T-C_2	224.262	1.529
C_2-C_2	224.262	1.529
C_T-H_T	constrained	1.090
C_2-H_2	constrained	1.090

Table S2. Harmonic angle potential (Eq. S3)

**Angles**	$\boldsymbol{K_{angle}}$ (kJ mol^{-1} rad^{-2})	$\boldsymbol{\Theta^0_{ijk}}$ (deg)
C_T-C_2-C_2	488.273	112.70
C_T-C_2-H_2	488.273	112.70
C_2-C_2-H_2	488.273	112.70
C_2-C_T-H_T	313.800	110.70
C_T-C_2-H_2	313.800	110.70
C_2-C_2-H_2	313.800	110.70
H_T-C_T-H_T	276.144	107.80
H_2-C_2-H_2	276.144	107.80

Table S3. Fourier dihedrals (Eq. S4)

**Dihedrals**	$\boldsymbol{C_1}$	$\boldsymbol{C_2}$	$\boldsymbol{C_3}$	$\boldsymbol{C_4}$
C_T-C_2-C_2-C_2	-0.305938	2.697394	-0.896807	0.74567
C_2-C_2-C_2-C_2	-0.305938	2.697394	-0.896807	0.74567

Table S4. Ryckaert-Bellemans dihedral potential (Eq. S5)

Dihedrals	C_0	C_1	C_2	C_3	C_4	C_5
C_X-C_X-C_X-H_X*	0.6276	1.8828	0	-2.5104	0	0
H_X-C_X-C_X-H_X*	0.6276	1.8828	0	-2.5104	0	0

* Type X represents either type 2 or T

1.2 Atomistic Model of Graphitic planes and CB's Core and Shell Particles

In the Figure S2, the structure of an atomistic graphitic plane, and the constituting CB primary particle molecules, named *core* and *shell*, are reported. More in detail, the core and shell molecules are composed of 48 and 336 atoms, respectively. For both core and shell, the carbon atoms at the edge of the molecule are bonded with hydrogen atoms, as can be seen from the figure. The hydrogen atoms are named H_b, while the carbon atom bonding a hydrogen atom is name C_b. All other carbon atoms, which are bonded exclusively with other carbons, are name C. All bonds involving hydrogen atoms have been constrained by using the LINCS algorithm.[9] The graphitic layer, representing an infinite and continues structure, is connected with itself, by the PBC condition. For this reason, there are not carbon atoms bonded with hydrogens. In that case, all atoms are considered of the type C. In overall, the OPLS-AA force-field is used for all bonded and non-bonded interactions (with a number of exclusions $n_{excl.} = 3$). In the following, the adopted force-field parameters are listed in Tables S5-S9.

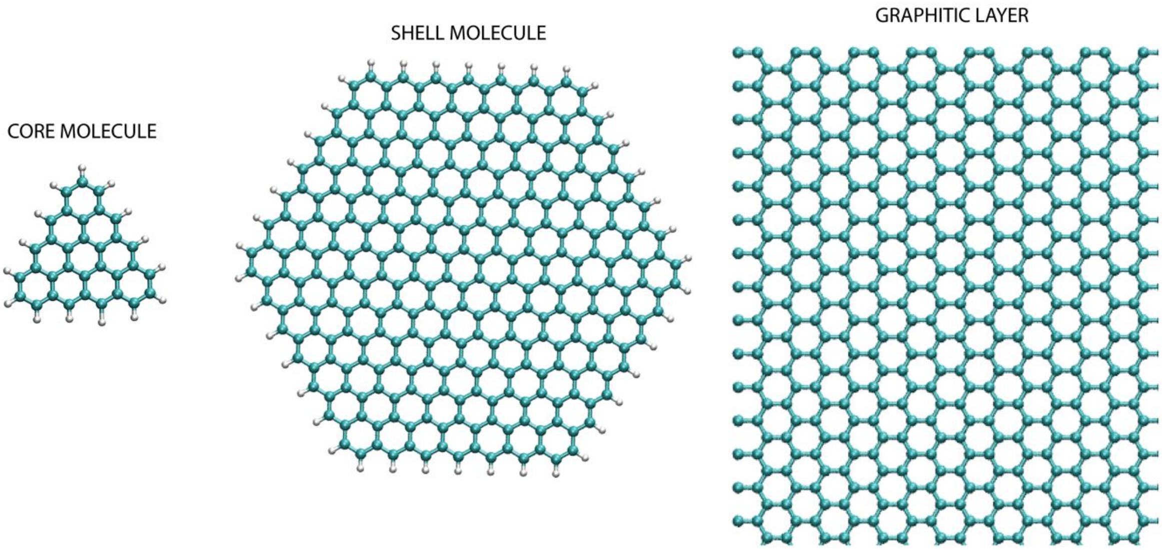

Figure S2. Chemical structure of core particle (on the left), shell particle (in the middle), and the graphitic layer (on the right).

Table S5. Lennard-Jones parameters and Coulomb charges (Eq. S1)

Atom Types	σ (Å)	ε (kJ mol^{-1})	q
C	0.355	0.29288	0
C_b	0.355	0.29288	-0.115
H_b	0.242	0.12552	0.115

Table S6. Harmonic bond potential (Eq. S2)

Bonds	K_{bond} (kJ mol^{-1} Å^{-2})	r_{ij}^0 (Å)
C-C	392.459	1.400
C_b-C	392,459	1.400
C_b-H_b	Constrained	1.080

Table S7. Harmonic angle potential (Eq. S3)

Angles	K_{angle} (kJ mol^{-1} rad^{-2})	Θ^0_{ijk} (deg)
C-C-C	527.184	120.00
C-C-C_b	527.184	120.00
C-C_b-C	527.184	120.00
C_b-C_b-C	527.184	120.00
H_b-C_b-C	292.88	120.00

Table S8. Ryckaert-Bellemans dihedral potential (Eq. S5)

Dihedrals	C_0	C_1	C_2	C_3	C_4	C_5
Cx-Cx-Cx-Cx*	30.334	0	- 30.334	0	0	0
H_b-Cx-Cx-H_b*	30.334	0	- 30.334	0	0	0
Cx-Cx-Cx-H_b*	30.334	0	- 30.334	0	0	0

* Type X represents either C or C_b

The improper dihedrals $V_{id}(\psi_{ijkl})$ have the following functional form:

$$V_{id}(\psi_{ijkl}) = \frac{1}{2}k_{\phi}(\phi_{ikjl} - \phi_o)^2 \qquad \text{(S6)}$$

Table S9. Improper dihedral potential (eq. S6)

Dihedrals	ϕ	k_{ϕ} (kJ/mol/rad^2)
H_b-C_b-C-C	180.0	10.46

1.3 Equilibration procedure for the atomistic systems

All atomistic simulations, including the PE polymer melts and the composite system of PE interacting with the graphitic layers, have been previously equilibrated by using the hPF approach. The equilibrated configurations of all the systems were obtained by using the OCCAM package[4] for the hPF MD, and following the procedure validated in the reference [5]. According to the procedure reported by De Nicola et al.[5], two subsequent hPF runs, increasing the level of description (reducing the mesh size from l = 0.4 nm to l = 0.2 nm), were performed.[4–6] The final stage of the procedure consists in the removing unphysical atom overlaps. To this aim, a short run (5000 steeps) of energy minimization by using the steepest descent algorithm is performed for all systems. Then, standard MD simulations were performed. As first, a short run (5 ns) in the NVT ensemble, followed by the production runs (in the NPT ensemble). For both, energy minimization and MD runs, the GROMACS package[8] is used. In Table S10, the composition of the simulated systems is reported.

Table S10. Composition of the systems and simulation details, for the all atom MD simulations in the NPT ensemble.

**System**	**No. of PE Chains**	**No. of Monomers in PE chains**	**Tot. No. Particles**	**Box size** [nm] (x,y,z)	**Time** (ns)	**T** (K)
PE	191	20	23302	6.276<br>5.599<br>5.793	100	293
PE	191	20	23302	6.815<br>6.500<br>6.291	180	550
PE	40	1072	257360	14.120<br>14.120<br>14.120	140	423
GR/PE	388	20	59342	5.880<br>6.542	240	550

1.4 Atomistic Simulation of the GR/PE System

At atomistic level, the interface between the polymer melt of PE and graphitic surface (miming the carbon black surface) has been simulated by using the graphitic layers (See Figure S2). Eight layers, in contact with low M.W. of PE (20 repeating units for chain) were simulated. In the panel A of Figure S3, a snapshot of the simulated system is reported, while, in the panel B of the same figure the mass density profile, calculated along the normal direction of the graphitic layers, is reported too. The initial set of coordinates is obtained by following the same procedure reported in the section 1.3.

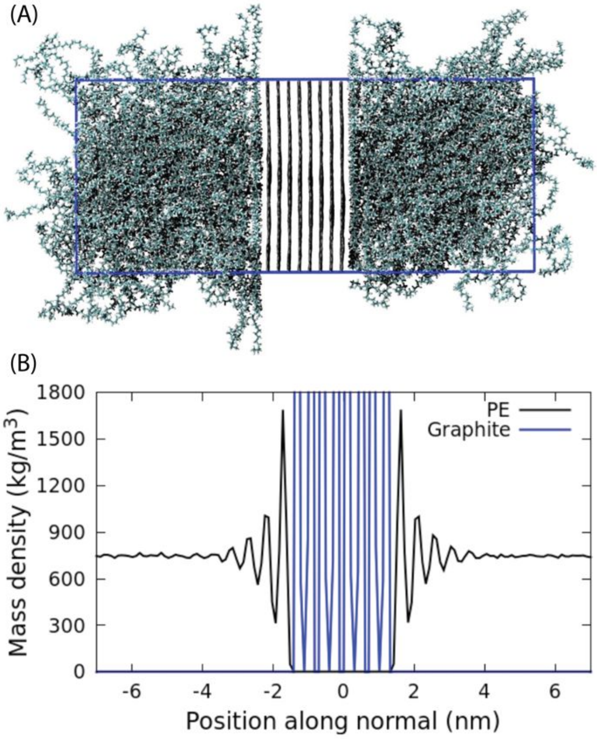

Figure S3. (A) snapshot of the GR/PE20 system. Eight graphitic layers are positioned at the middle of the box. Each graphitic layer contains 864 carbon atoms. In total, 6912 carbon atoms are used to represents the graphitic layers. (B) Mass density profile of PE and GR calculated along the normal direction to graphitic planes. The profiles have been averaged over the last 40 ns of production run.

1.5 Computational Details for Atomistic Simulations

For all the atomistic simulations in the NVT and NPT ensembles, the temperature was controlled by using the velocity rescale algorithm with a coupling constant τ_T = 0.02 ps. In the NPT ensemble runs the pressure was held constant at 1.013 bar using the Berendsen algorithm with a coupling constant τ_P = 0.2 ps. A time step of 2 fs was employed in all simulations. A cutoff distance of 1.1 nm was used for both van der Waals and Coulomb interactions. The electrostatic interactions were treated by using the Ewald summation method. The LINCS constraint algorithm[9] was employed to fix all the carbon-hydrogen distances.

2. Coarse-Grained Models

2.1 Model of Core and Shell CG molecules of Carbon Black (CB)

The non-bonded interactions of the proposed CG model for core and shell molecules, composing the CB primary particle, are based on the Lennard-Jones potential (eq. S7).

$$V(r_{ij}) = 4\varepsilon_{ij}\left[\left(\frac{\sigma_{ij}}{r_{ij}}\right)^{12} - \left(\frac{\sigma_{ij}}{r_{ij}}\right)^{6}\right]. \quad (S7)$$

In order to keep the model as simple as possible, a single pair of LJ parameters σ and ϵ is used for both molecules (core and shell). The parameterization of σ and ϵ for the CG model is based on the reproduction of the reference atomistic LJ potential. According to the CG mapping scheme, see the Figure S4, the LJ potential of two CG core molecules is calculated at different distance (d). In the equivalent atomistic system, the distance (d) is changed between two pairs of core molecules (the distance between each pair of atomistic molecules is fixed at 0.73 nm). In Figure S3, according to our choice for the parameterization, the best matching of LJ potential for core and shell CG molecules is reported. Finally, the **σ** = 0.635 nm and **ε** = 8.55 kJ/mol are used for both core and shell molecules composing the carbon black primary particle.

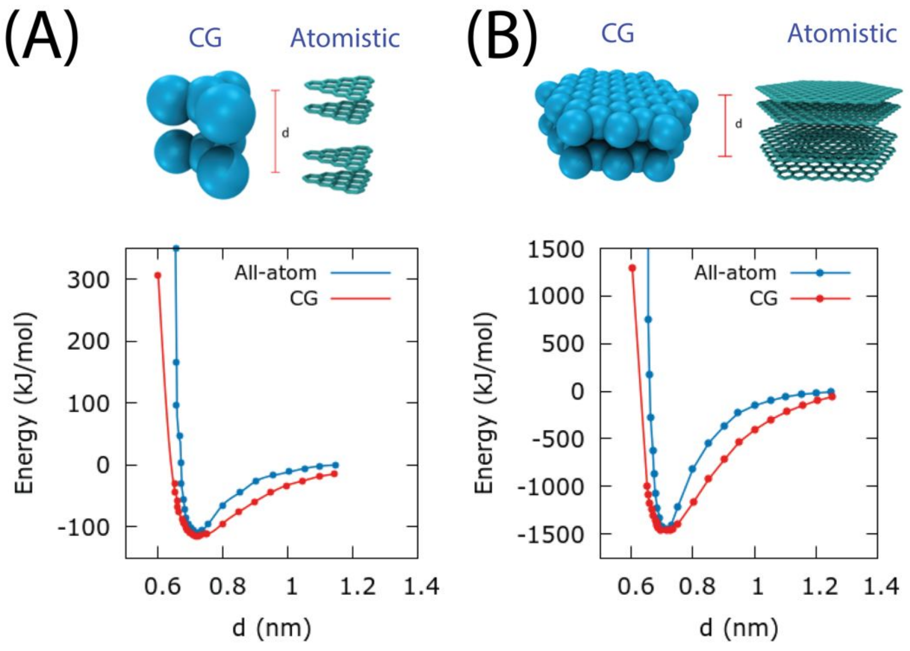

Figure S4. Comparison between the LJ potential calculated for atomistic and CG models in the case of core (panel A) and shell (panel B) particles.

In the following, the parameters for the bonded interactions of core and shell molecules are reported (Tables S11-S13). For both molecules, the CG bead is named G. In Figure S5 and Figure S6, the scheme of the bond and angle for core and shell CG molecules is reported.

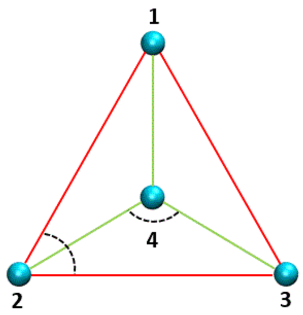

Figure S5. Schematic view of core particle. Light blue spheres represent centers of mass of beads. Red and green lines represent bond connections between CG beads. All angles of 60° (e.g. 1-2-3) and 120° (e.g. 2-4-3) are specified in the topology.

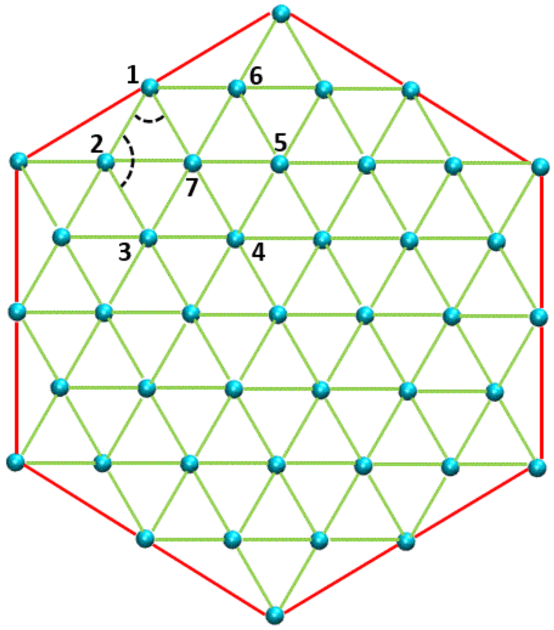

Figure S6. Schematic view of core particle. Light blue spheres represent centers of mass of beads (G). Red and green lines represent bond connections between CG beads. All the angles of 60° (e.g. 1-2-7) and 120° (e.g. 1-2-3), and all the dihedral quadruplets (e.g. 1-2-3-4, 2-3-4-5, 3-4-5-6, 4-5-6-1, 5-6-1-2, 6-1-2-3) are specified in the topology.

Table S11. Harmonic bond potential (Eq. S2)

**Bonds**	K_{bond} (kJ mol^{-1} Å^{-2})	r_{ij}^{0} (Å)
G-G (green)	3.92	7.380
G-G (red)	3.92	4.260

The angle potential for the CG core and shell molecules has the functional form:

$$V_a(\theta_{ijk}) = \frac{1}{2} K_{angle}(\cos(\theta_{ijk}) - (\theta_0))^2 \qquad \text{(S8)}$$

where θ_0 is the equilibrium angle and K_{angle} is the potential constant. In Table S12, the parameters for the harmonic angle potential are listed. In particular, according to the schemes reported in the Figure S4 and S5, two angles are described, a minor (60º) and a major (120º).

Table S12. Harmonic angle potential (Eq. 2)

**Angles**	K_{angle} (kJ mol^{-1} rad^{-2})	Θ^{0}_{ijk} (deg)
G-G-G (minor)	8.0	120.00
G-G-G (major)	8.0	60.00

Table S13. Ryckaert-Bellemans dihedral potential (Eq. S5)

**Dihedrals**	C_0	C_1	C_2	C_3	C_4	C_5
G-G-G-G	30.334	0	- 30.334	0	0	0

2.2 Annealing Procedure to build the CB primary particle

The initial configuration of the CB primary particle has been obtained by following the procedure reported in this section. As fist step, the core of the CB primary particle has been built by imposing geometrical constraints for the packing of core molecules. In particular, according to the density of 2 g/cm^3, 796 core molecules were placed inside a sphere of radius R = 5 nm. Then, the energy of the initial configuration for the core of CB has been minimized using a steepest descent algorithm (150 steps with position restraints on molecules, followed by additional 150 ps without position restraints). Then, the structure was equilibrated by performing two consecutive runs of 15 ns in the NVT ensemble at 500K. The final configuration of the core has been used to build the whole CB primary particle. In particular, 626 shell particles have been geometrically constrained in a spherical shell, next to the core

assembly, having as minimum radius $R_{min} = 5$ nm and maxim radius $R_{max} = 10$ nm. The geometrical constraints and packing of the molecules were done with the Packmol code.[10] As in the case of the core, the initial structure has been energy minimized by using the steepest descent algorithm (two consecutive runs: 2 x 600 ps). Then, successive annealing runs, each one of 15 ns, were performed in the NVT ensemble. In Figure S7, the applied annealing pathway is reported.

For all simulations, the temperature was controlled by using the velocity rescale algorithm with a coupling constant τ_T = 0.2 ps. A time step of 3 fs was employed in all simulations. A cutoff distance of 2.1 nm was used for both van der Waals and Coulomb interactions. The non-bonded interactions are excluded between first and fourth neighbours.

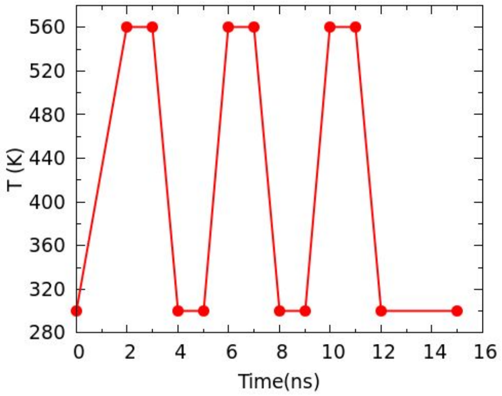

Figure S7. Variation of the temperature during the simulated annealing simulation.

2.3 Aggregation of 9 and 18 CB primary particles

The aggregation of both aggregates composed of 9 and 18 CB primary particles have been obtained by placing a single CB particle at the centre of the simulation box. Then, the 2th CB particle was added at distance $d_{ij} = 2R + 1.0$ nm, where $R = 10$ nm. The 3rd one was added in a similar way by checking if the geometrical constraint (d_{ij}) were satisfied for each pair of CBs. The remaining CB particles were added by following the same criteria. Since the initial positions of CBs have been chosen to avoid overlaps between shell particles of each CBs, a short MD simulation was performed (100 ns of MD simulation, in NVT ensemble at 500 K) to allow CBs to assembly. In Table S14, the composition of all simulated systems is reported.

Table S14. Composition of the CB CG systems. Production run after the assembly procedure. The simulations have been performed in the NVT ensemble.

No. of CB Primary Particles	No. of Core Molecules	No. of Shell Molecules	Tot. No. Particles	Box size (nm) [x = y = z]	Time (ns)	T (K)
1	796	626	30102	50	100	550
9	7164	5634	270918	150	10	550
18	14328	11268	541836	200	10	550

2.4 Graphitic Planes (GR)

The CG representation of the graphitic layers uses the same mapping scheme as for core and shell molecules of the CB primary particles. As direct consequence of that choice, the same distances between neighbouring beads (0.426 and 0.738 nm) are found. In particular, each GR CG layer corresponds to two atomistic layers. The bonded and non-bonded interactions among the beads belonging GR layers are not computed. During the hPF MD simulations, all beads belonging GR are kept frozen by excluding them from the integration algorithm. In addition, for the hPF CG model of GR, two different bead type are considered, L1 and L2, respectively. The interaction of PE with both bead types is described in the next section. In Figure S8, a representation of the GR layers is reported.

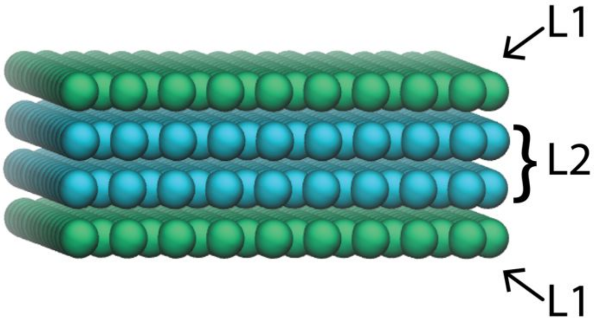

Figure S8. CG representation of GR graphitic layers. The two external layers (in green) are composed of beads of type L1, while the layers in the middle (in light blue) are composed of L2 bead type.

2.5 χ (hPF) Parameterization

Differently from the CG model of CB primary particle based on pair potentials (the non-bonded interactions are evaluated via LJ potential), in the case of hPF CG models the non-bonded interactions are treated in the approximation of mean-field. The interactions between two different bead types (KK') are calculated by using the effective interaction parameter $\chi_{KK'}$. The interacting χ parameters describing the interaction between the polymer PE and the beads of core and shell molecules, and with the graphitic layers, have been parameterized by using reference atomistic simulations. For the parameterization of χ, we used the graphitic layers (8 layers in the case of atomistic simulation, and 4 layers in the case of CG system) interacting with PE polymer melt. The composition of the all-atom reference system and the analogous CG are reported in Table S15. Based on the best matching between atomistic and CG density profiles, the χ parameters have been tuned. In particular, as reported in the main text of the manuscript, the CG model of core, shell and graphitic layers have two different bead types: L1, and L2. The first, (L1) has a repulsive interaction with the polymer bead type PE, while the second one (L2) has, instead, an attractive interaction with PE. Between the two bead types L2 and L1, there is no interaction. In the Figure S9, the density profiles calculated for the reference atomistic system, and for different χ parameters are reported. In Table S16, the final parameter list for χ is reported.

Table S15. Composition of the systems and simulation details, for the all atom and CG MD simulations in the NVT ensemble.

System	No. of PE Chains	No. of Monomers in PE chains	Tot. No. Particles	Box size (nm) [x,y,z]	Time (ns)	T (K)
Graphite/PE (All-atom)	388	20	59342	5.880<br>6.542<br>15.550	240	550
Graphite/PE (CG)	388	5	3024	5.880<br>6.542<br>15.550	600	550

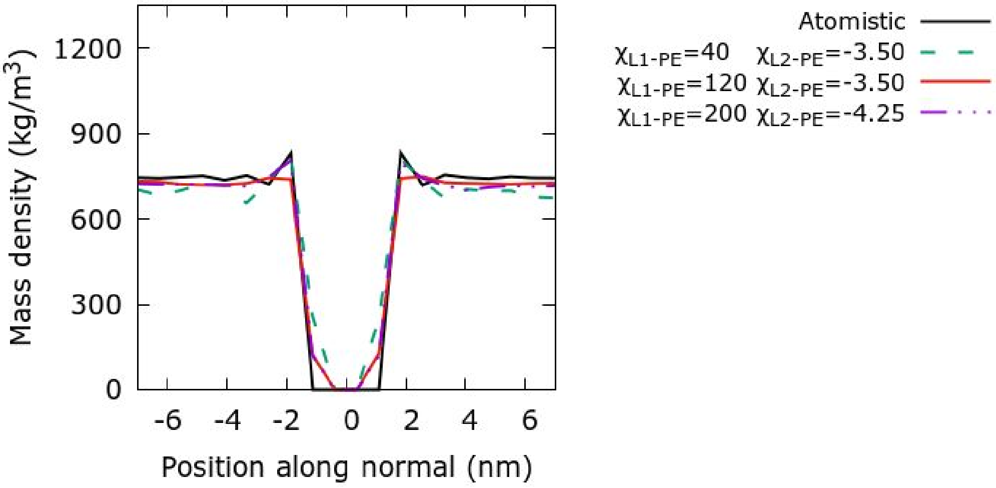

Figure S9. Polymer density profiles used for the parametrization. Atomistic reference profile (black curve) and three pairs of $\chi_{KK'}$ interaction parameters are compared.

Table S16. Interaction matrix $\tilde{\chi}_{KK'} = \chi_{KK'} \cdot RT$ (kJ mol^{-1}) for CG PE/Graphite system.

$\tilde{\chi}_{KK'}$	**L1**	**L2**	**PE**
**L1** (repulsive)	0	0	200
**L2** (attractive)	0	0	- 4.25
**PE**	200	- 4.25	0

2.6 PE Model

The mapping scheme for the CG model of PE was chosen to match as much as possible the length scale of the bond length of CG model of graphitic planes and, core and shell molecules, as reported in the Figure 3A and 3B of the main text. In the following, the functional form and parameters used for the bonded interaction of PE CG model are reported. In particular, the bonds and the angles are treated by using the harmonic potential (eqs. S9 and S10):

$$V_b(\mathbf{r}_{ij}) = \frac{1}{2} K_{bond} (\mathbf{r}_{ij} - \mathbf{r}_{ij}^0)^2 \qquad \text{(S9)}$$

$$V_a(\theta_{ijk}) = \frac{1}{2} K_{angle} (cos(\theta_{ijk}) - \cos(\theta_0))^2 \qquad \text{(S10)}$$

where K_{bond} and K_{angle} are the constant parameters and $\mathbf{r}_{ij}^0$ and θ_0 are the equilibrium bond length and angle, respectively. In the Table S17 and S18, the used parameters are reported.

Table S17. bond potential parameters (Eq. S9)

**Bond**	K_{bond} (kJ mol^{-1} nm^{-2})	r_{ij}^0 (nm)
C-C	400.0	0.715

Table S18. angle potential parameters (Eq. S10)

**Angle**	K_{bond} (kJ mol^{-1} rad^{-2})	θ_0 (deg.)
C-C-C	7.5	165.0

3 Additional Analysis

3.1 Interpenetration Length

The interpenetration length (IL) between the solid surfaces (CB or GR) and the PE is calculated by evaluating the overlap integral by using the following equation:

$$IL = \int dz \cdot \rho_{Surface}(z) \cdot \rho_{PE}(z) \qquad (S11)$$

Where $\rho_{Surface}(z)$ and $\rho_{PE}(z)$ are the normalized density values, as function of the distance from the surface (z), calculated for PE and the surface. The normalization is calculated by considering the bulk value of each species.[11] In particular, ρ_{PE} is normalized with respect to the bulk density of PE (0.726 g/cm^3), and $\rho_{Surface}$ is normalized with respect to the bulk density of the CB (2.0 g/cm^3).

Table S19. Interpenetration Length (IL) calculated for mono- and bi- disperse systems.

**PE**	**Surface**	***IL*** (nm)
1072	GR	2.00
5880	GR	2.38
10696	GR	2.24
1072	CB	2.79
5880	CB	2.80
10696	CB	1.98

3.2 Comparison of Diffusion coefficients

The dynamics of CG models is, usually, faster than the corresponding atomistic systems and this is a further advantage in terms of computational costs. This is caused by smoother energy landscapes because of larger particle sizes and, in the present case, the use of smooth potentials based on density fields. Comparisons between dynamical properties calculated with a CG model and corresponding all-atom models determined properties can give an evaluation of the dynamics. In particular, the model dynamics can be rescaled by multiplying the actual value of the time step in the CG model Δt_{CG} by a scaling factor τ_{CG} due to the smoothness of the potentials. The comparison between diffusion coefficients calculated from the CG models and atomistic simulations can be used to evaluate the scaling factor. For the present case, the

behaviour of the diffusion coefficient of the center of mass of a PE chain as function of molecular weight has been obtained from the proposed CG model and experimental values are compared in Figure S10 and Table S20. For smaller chains PE1072 a scaling factor τ_{CG} ~ 70 is obtained, while for the longer PE10796 a larger value of τ_{CG} ~ 700 is found. It is worth noting that this scaling factor is not universal and can be related only to the process of chain diffusion. Moreover, the evaluation of end to end chain relaxation times gives a quantitative estimation of the time required to obtain an independent chain configuration. For the shortest PE20 chains the relaxation behavior is similar (τ = 0.128 ± 0.003 ns and τ = 0.123 ± 0.004 ns, for the all-atom and CG models, respectively), while for the longer PE1072 the CG model shows a relaxation time about three times faster than the corresponding all atom model (τ = 2.52 ± 0.07 ns and τ = 0.80 ± 0.08 ns, for the all-atom and CG models, respectively).

Table S20. Values of calculated and experimental[12] diffusion coefficients of CG PE chains. The diffusion coefficient have been calculated at T = 468K.

**No. of Repeating Units**	$D_{calculated}$ (cm^2/s)	$D_{experimental}$ (cm^2/s)	$D_{calculated}/D_{experimental}$
20	4.1e-06	6e-06	0.68
1072	1.6e-07	2.2e-09	72
5880	3.5e-08	7.7e-11	450
10696	1.6e-08	2.3e-11	700

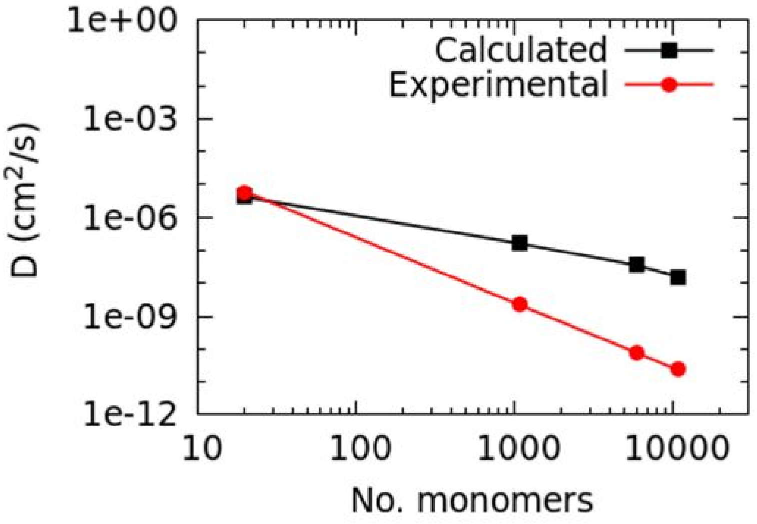

Figure S10. Values of calculated and experimental[12] diffusion coefficients of CG PE chains, at T = 468 K

3.3 End to end autocorrelation functions (ACF)

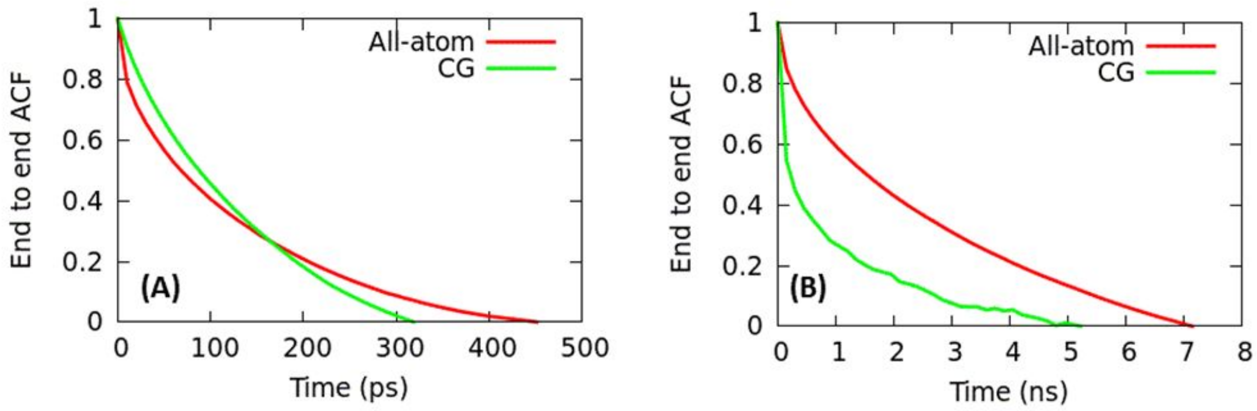

Figure S11. Comparison of ACFs from all atom and CG models for PE20 and PE1072.

$$\tau_\alpha = \int_0^\infty exp\left[-\left(\frac{t}{\alpha}\right)^\beta\right]dt = \frac{\alpha}{\beta}\Gamma\left(\frac{1}{\beta}\right) \quad \text{(S12)}$$

Table S21. Fitting Parameter and calculated τ for ACFs.

**System**	**α**	**β**	**τ_α (calculated)** [ns]
PE 20 (All-atom)	0.106 ± 0.02	0.90 ± 0.02	0.128 ± 0.003
PE20 (CG)	0.104 ± 0.003	0.92 ± 0.02	0.123 ± 0.004
PE 1072 (All-atom)	2.18 ± 0.04	0.93 ± 0.02	2.52 ± 0.07
PE 1072 (CG)	0.66 ± 0.05	0.91 ± 0.07	0.80 ± 0.08
PE 10696 (CG)	8.87 ± 0.55	0.92 ± 0.06	9.24 ± 0.86

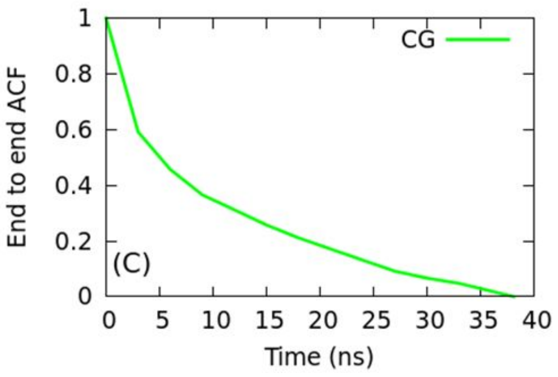

Figure S12. ACFs from CG model CB/PE10696

3.4 Effect of Interface Mode on CB/PE Density Profile.

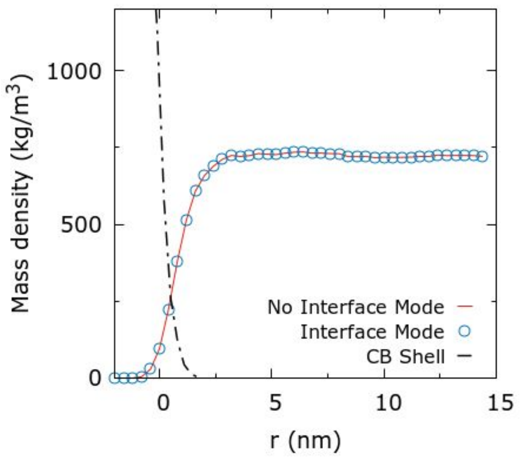

Figure S13. Density Profiles of PE1072 at interface with a single CB particle. The position of the origin for the CB particle is located at a distance of 10 nm (i.e. the radius of the CB particle) from the centre of mass of the particle. The Δt_{update} was set equal to 3 ps. When used, IM update was set equal to 3 ps.

References

(1) Salerno, K. M.; Agrawal, A.; Peters, B. L.; Perahia, D.; Grest, G. S., Dynamics in Entangled Polyethylene Melts. *Eur. Phys. J. Spec. Top.* **2016**, *225*, 1707.

(2) Güryel, S.; Walker, M.; Geerlings, P.; De Proft, F.; Wilson, M. R., Molecular Dynamics Simulations of the Structure and the Morphology of Graphene/Polymer Nanocomposites. *Phys. Chem. Chem. Phys.* **2017**, *19*, 12959.

(3) Ewen, J. P.; Gattinoni, C.; Thakkar, F. M.; Morgan, N.; Spikes, H. A.; Dini, D., A Comparison of Classical Force-Fields for Molecular Dynamics Simulations of Lubricants. *Materials.* **2016**, *9*, 651.

(4) Zhao, Y.; De Nicola, A.; Kawakatsu, T.; Milano, G., Hybrid Particle-Field Molecular Dynamics Simulations: Parallelization and Benchmark. *J. Comput. Chem.* **2012**, *33*, 868–880.

(5) De Nicola, A.; Kawakatsu, T.; Milano, G., Generation of Well-Relaxed All-Atom Models of Large Molecular Weight Polymer Melts: A Hybrid Particle-Continuum Approach Based on Particle-Field Molecular Dynamics Simulations. *J. Chem. Theory Comput.* **2014**, *10*, 5651.

(6) De Nicola, A.; Correa, A.; Milano, G.; La Manna, P.; Musto, P.; Mensitieri, G.; Scherillo, G., Local Structure and Dynamics of Water Adsorbed in Polyetherimide: A Hydrogen Bonding Anatomy, *J. Phys. Chem.* **2017**, *121*, 3162–3176.

(7) De Nicola, A.; Avolio, R.; Della Monica, F.; Gentile, G.; Cocca, M.; Capacchione, C.; Errico, M. E.; Milano, G., Rational Design of Nanoparticle/Monomer Interfaces: A Combined Computational and Experimental Study of in Situ Polymerization of Silica Based Nanocomposites. *RSC Adv.* **2015**, *5* (87), 71336–71340.

(8) Berendsen, H. J. C.; Van Der Spoel, D.; Van Drunen, R., GROMACS: A Message-Passing Parallel Molecular Dynamics Implementation PROGRAM SUMMARY Title of Program: GROMACS Version 1.0. *Comput. Phys. Commun.* **1995**, *91*, 43–56.

(9) Hess, B.; Bekker, H.; Berendsen, H. J. C.; Fraaije, J. G. E. M. LINCS: A Linear Constraint Solver for Molecular Simulations. *J. Comput. Chem.* **1997**, *18* (12), 1463–1472.

(10) Martínez, L.; Andrade, R.; Birgin, E. G.; Martínez, J. M. Packmol: A Package for Building Initial Configurations for Molecular Dynamics Simulations. *J. Comput. Chem.*

2009, *30* (13), 2157–2164.

(11) Galuschko, A.; Spirin, L.; Kreer, T.; Johner, A.; Pastorino, C., Wittimer, Baschnagel, J., Frictional Forces between Strongly Compressed, Nonentangled Polymer Brushes: Molecular Dynamics Simulations and Scaling Theory. *Langmuir* **2010**, *26* (9), 6418–6429.

(12) Pearson, D. S.; Ver Strate, G.; Von Meerwall, E.; Schilling, F. C.; Viscosity and Self-Diffusion Coefficient of Linear Polyethylene. *Macromoleules* **1987**, *20*, 1133.
